# Investigating *LMNA*-Related Dilated Cardiomyopathy Using Human Induced Pluripotent Stem Cell-Derived Cardiomyocytes

**DOI:** 10.3390/ijms22157874

**Published:** 2021-07-23

**Authors:** Yuval Shemer, Lucy N. Mekies, Ronen Ben Jehuda, Polina Baskin, Rita Shulman, Binyamin Eisen, Danielle Regev, Eloisa Arbustini, Brenda Gerull, Mihaela Gherghiceanu, Eyal Gottlieb, Michael Arad, Ofer Binah

**Affiliations:** 1 Department of Physiology, Biophysics and Systems Biology, Rappaport Faculty of Medicine and Rappaport Research Institute, Technion—Israel Institute of Technology, Haifa 31096, Israel; yyshemer@gmail.com (Y.S.); lucymekies@yahoo.fr (L.N.M.); ronenbeje@gmail.com (R.B.J.); polinabaskin@campus.technion.ac.il (P.B.); rita.shulman@gmail.com (R.S.); binyae@campus.technion.ac.il (B.E.); r.danielle@campus.technion.ac.il (D.R.); 2 Department of Biotechnology, Technion—Israel Institute of Technology, Haifa 3200003, Israel; 3 Centre for Inherited Cardiovascular Diseases, IRCCS Foundation, Policlinico San Matteo, 27100 Pavia, Italy; e.arbustini@smatteo.pv.it; 4 Comprehensive Heart Failure Center and Department of Internal Medicine I, University Hospital Würzburg, 97080 Würzburg, Germany; gerull_b@ukw.de; 5 Victor Babes National Institute of Pathology, 050096 Bucharest, Romania; mgherghiceanu@yahoo.com; 6 Department of Cell Biology and Cancer Science, Rappaport Faculty of Medicine, Technion—Israel Institute of Technology, Haifa 31096, Israel; e.gottlieb@technion.ac.il; 7 Leviev Heart Center, Sheba Medical Center, Ramat Gan 52621, Israel; michael.arad@sheba.health.gov.il; 8 Sackler Faculty of Medicine, Tel Aviv University, Tel Aviv 6997801, Israel

**Keywords:** *LMNA*, dilated cardiomyopathy, iPSC-CMs, electrophysiology, arrhythmia

## Abstract

*LMNA*-related dilated cardiomyopathy is an inherited heart disease caused by mutations in the *LMNA* gene encoding for lamin A/C. The disease is characterized by left ventricular enlargement and impaired systolic function associated with conduction defects and ventricular arrhythmias. We hypothesized that *LMNA*-mutated patients’ induced Pluripotent Stem Cell-derived cardiomyocytes (iPSC-CMs) display electrophysiological abnormalities, thus constituting a suitable tool for deciphering the arrhythmogenic mechanisms of the disease, and possibly for developing novel therapeutic modalities. iPSC-CMs were generated from two related patients (father and son) carrying the same E342K mutation in the *LMNA* gene. Compared to control iPSC-CMs, *LMNA*-mutated iPSC-CMs exhibited the following electrophysiological abnormalities: (1) decreased spontaneous action potential beat rate and decreased pacemaker current (I_f_) density; (2) prolonged action potential duration and increased L-type Ca^2+^ current (I_Ca,L_) density; (3) delayed afterdepolarizations (DADs), arrhythmias and increased beat rate variability; (4) DADs, arrhythmias and cessation of spontaneous firing in response to β-adrenergic stimulation and rapid pacing. Additionally, compared to healthy control, *LMNA*-mutated iPSC-CMs displayed nuclear morphological irregularities and gene expression alterations. Notably, KB-R7943, a selective inhibitor of the reverse-mode of the Na^+^/Ca^2+^ exchanger, blocked the DADs in *LMNA*-mutated iPSC-CMs. Our findings demonstrate cellular electrophysiological mechanisms underlying the arrhythmias in *LMNA*-related dilated cardiomyopathy.

## 1. Introduction

The *LMNA* gene encodes for the nuclear intermediate filament proteins A-type lamins [1]. The two main products of this gene, lamin A and lamin C, result from alternative splicing and are expressed in most differentiated somatic cells [2,3]. In addition to their structural role in maintaining nuclear stability, these proteins are involved in a variety of cellular pathways such as chromatin organization, nucleoskeleton-cytoskeleton connection, gene expression and DNA replication and repair [4,5,6,7]. Mutations in the *LMNA* gene cause a diverse group of diseases known as laminopathies which include, among others, dilated cardiomyopathy (DCM) type 1A, Emery–Dreifuss muscular dystrophy type 2 and type 3, limb–girdle muscular dystrophy type 1B, Dunnigan-type familial partial lipodystrophy, Charcot–Marie–Tooth disease type 2B1 and Hutchinson–Gilford progeria syndrome (HGPS) [8]. *LMNA*-related DCM is suggested to account for ~5–8% of familial DCM, and is characterized by left ventricular dilation, impaired systolic function, conduction defects and arrhythmias [9,10]. Consequently, patients are at risk for heart failure and sudden cardiac death [10,11]. As in the case of other inherited cardiac disorders, insufficient access to human-sourced cardiomyocytes has hampered attempts to understand cellular mechanisms underlying the disease. Particularly, due to the large inter-species variations respecting cardiac electrophysiology, human cardiomyocytes are very useful for investigating the arrhythmogenic substrate of *LMNA* cardiomyopathy. Therefore, we hypothesized that *LMNA*-mutated patients’ induced Pluripotent Stem Cell-derived cardiomyocytes (iPSC-CMs) display electrophysiological abnormalities underlying the disease, thus constituting a suitable tool in furthering our knowledge of the arrhythmogenic mechanisms of the disease, and possibly for developing novel therapeutic modalities. Accordingly, iPSC-CMs were generated from two related (father and son) patients carrying the same mutation in the *LMNA* gene. In support of our hypothesis, we found that *LMNA*-mutated iPSC-CMs exhibit electrophysiological abnormalities including arrhythmias, and abnormal response to conditions causing elevated [Ca^2+^]_i_ levels. Importantly, the application of KB-R7943, a selective inhibitor of the reverse-mode of the Na^+^/Ca^2+^ exchanger (NCX), eliminated the DADs in *LMNA*-mutated iPSC-CMs. Additionally, compared to healthy control (Control) iPSC-CMs, *LMNA*-mutated iPSC-CMs exhibited nuclear morphological irregularities as well as gene expression alterations including genes encoding for major Ca^2+^ handling proteins.

## 2. Results

### 2.1. The LMNA-Mutated Patients and iPSCs Characteristics

**Figure 1:**
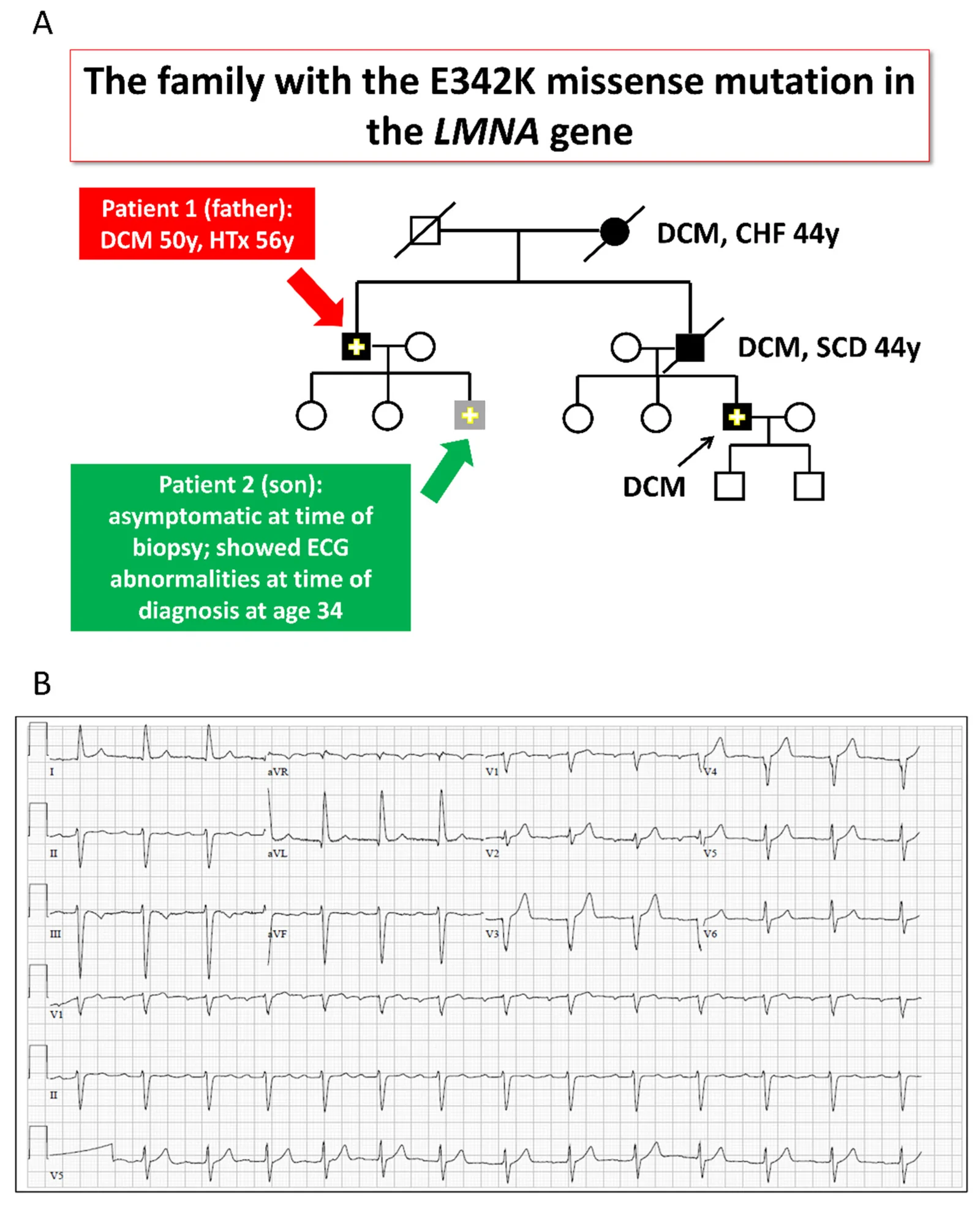
The *LMNA*-mutated patients. (**A**) Pedigree of the family with the *LMNA* gene mutation. Solid symbols: affected individuals; Solid symbols with a “+”: an affected individual with a proven mutation; Open symbols: clinically unaffected; Crossed symbol: deceased. Red arrow: a 63-year-old (at time of biopsy) DCM male patient, who underwent heart transplantation at age 56 for his severe heart failure (ejection fraction of 19%). His brother died at the age of 44 from the same disease, while awaiting heart transplantation. Green arrow: The 35-year-old son (at time of biopsy) of the 63-year-old patient was asymptomatic, but showed ECG abnormalities at the time of diagnosis at age 34. Biopsies were obtained from both the father and son. (**B**) Twelve-lead ECG of the son at age 34 demonstrating first-degree AV block and left anterior fascicular block.

**Figure 2:**
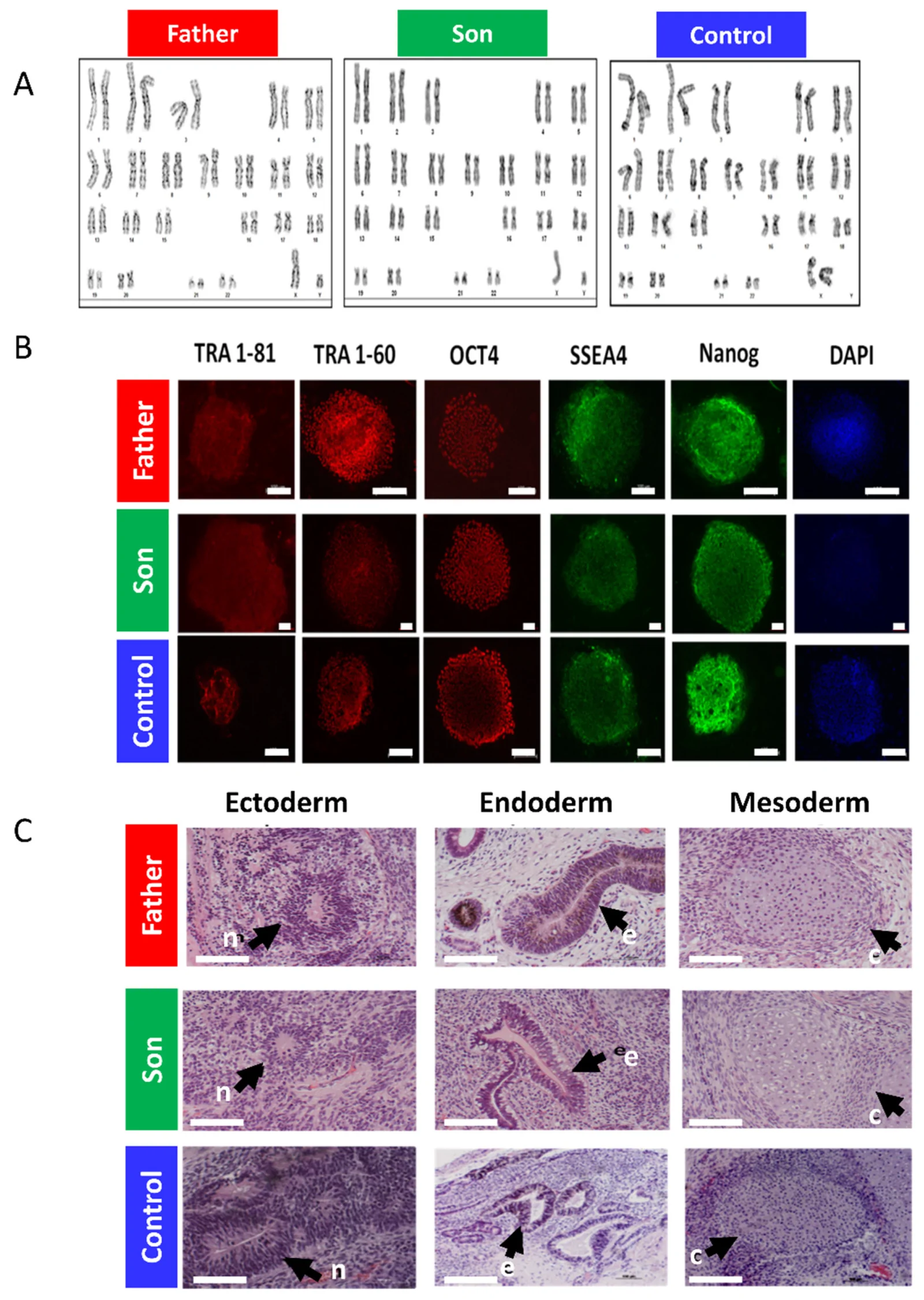
The *LMNA*-mutated patients iPSCs characteristics. (**A**) Father, son and control iPSCs showing normal karyotype. (**B**) Pluripotency staining of *LMNA*-mutated and control iPSCs. Immunofluorescence staining of typical pluripotent markers Tra1-81 (red), Tra1-60 (red), Oct4 (red), SSEA4 (green) and Nanog (green) in iPSCs generated from father, son and control. Nuclei are stained with DAPI (blue). Scale bar represents 100 µm. (**C**) Teratoma histological analysis obtained from in vivo differentiated *LMNA*-mutated and control iPSCs. Teratomas were obtained from SCID-NOD mice injected with either father, son or control iPSCs which differentiated in vivo into derivatives of all three germ layers: ectoderm, endoderm and mesoderm. n—Neuronal tissue represents ectodermal lineage. e—Endodermal epithelium with prominent mucus-producing cells representing endoderm formation. c—Cartilage as well as the chondrocyte area and muscle representing mesoderm formation. Scale bar represents 100 µm.

Four members of the *LMNA*-mutated family (3 men and 1 woman) manifested late-onset familial DCM (Figure 1A). The proband (subject II-1, Figure 1A) presented heart failure and DCM at age 50 in association with ventricular tachycardia and intraventricular conduction defect. The subject underwent heart transplantation at age 56 and is doing well since then. Both his mother and brother suffered from DCM, dying from heart failure and sudden cardiac death, respectively (subjects I-2 and II-4, Figure 1A). The brother’s son (subject III-6, Figure 1A) was diagnosed with DCM and moderate left ventricular dysfunction after presenting with sinus bradycardia, second-degree atrioventricular block and ventricular arrhythmia. An E342K *LMNA* mutation was found in the proband and confirmed in III-6. The proband’s son (subject III-3, Figure 1A), also a mutation carrier, had a normal echo-Doppler but first-degree atrioventricular block and left anterior fascicular block on his ECG at age 34 (Figure 1B). At 44, he remains asymptomatic with no change in his condition, being treated by ramipril. The father and son’s fibroblasts were reprogrammed using the STEMCCA cassette, and patient-specific iPSC clones were generated: 16.5 and 21.10 (father and son, respectively), as described in Section 4 below. Sequence analysis performed on the PCR products demonstrated the presence of the E342K mutation in both father and son iPSC clones (Figure 1C). The father and son iPSCs had a normal karyotype (Figure 2A), expressed the embryonic markers Oct4, Sox2, SSEA4, TRA1-60, TRA1-81 and NANOG (Figure 2B) and were able to differentiate into the 3 germ layers (ectoderm, mesoderm and endoderm) as shown by the teratoma assay (Figure 2C). Exome sequencing of father and son iPSC DNA identified no additional pathogenic variants in the established cardiomyopathy and arrhythmia genes. In addition to variants classified as likely benign, we found two rare variants of uncertain significance; one shared between the father and son and one carried only by the father (Tables S1 and S2, Supplementary Materials). Of note, there are inconclusive notions in the literature regarding the relevance of this shared variant of uncertain significance (MAF 0.0027) in the CAV3 gene c.233C > T (p.Thr78Met), which are beyond the scope of the current study [12,13,14,15].

### 2.2. Electrophysiological Abnormalities and Arrhythmias in LMNA-Mutated iPSC-CMs

As *LMNA*-related cardiomyopathy is associated with arrhythmias and conduction defects, we investigated whether the mutated iPSC-CMs present electrophysiological abnormalities and arrhythmias resembling the clinical settings.

#### 2.2.1. Decreased Spontaneous Beat Rate and I_f_ in LMNA-Mutated Cardiomyocytes

**Figure 3:**
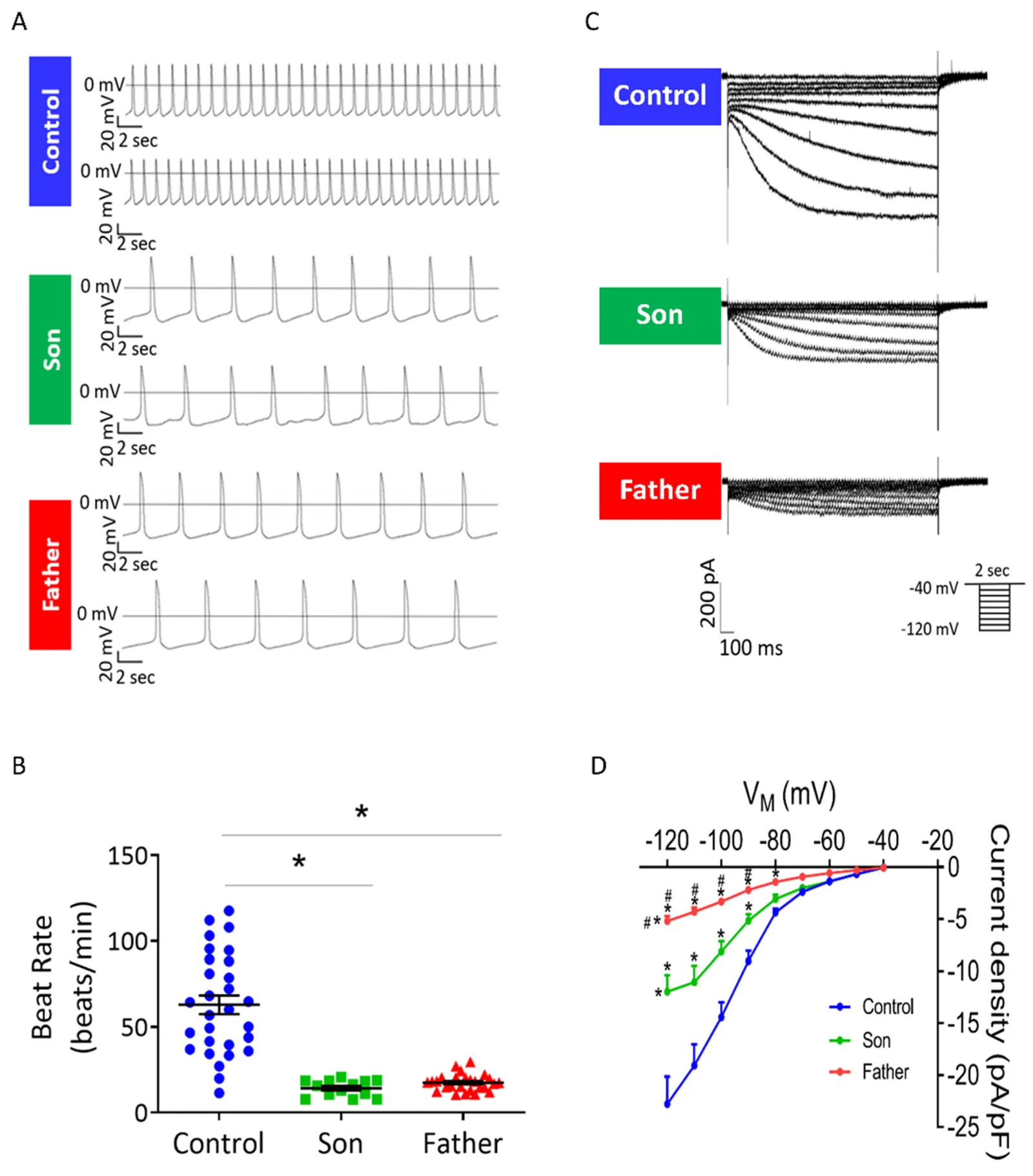
Spontaneous beat rate and I_f_ in control, father and son iPSC-CMs. (**A**) Representative spontaneous action potentials recorded from control, father and son iPSC-CMs. (**B**) Mean beat rate of control, father and son iPSC-CMs. control (FSE-5 m and 24.5), *n* = 30; son, *n* = 14; father, *n* = 27. One-way ANOVA was performed followed by Holm–Sidak post hoc analysis. * *p* < 0.05. (**C**) Representative recordings of I_f_ in control, father and son iPSC-CMs. (**D**) I_f_ current–voltage (I–V) relations in control and *LMNA*-mutated iPSC-CMs. Control (FSE-5 m), *n* = 8; son, *n* = 10; father, *n* = 18. Two-way ANOVA was performed followed by Holm–Sidak post hoc analysis. * *p* < 0.05 vs. control; # *p* < 0.05 vs. son.

A key electrophysiological abnormality in *LMNA* iPSC-CMs is markedly slower automaticity compared to control iPSC-CMs. As illustrated by the representative action potentials (Figure 3A) and the summary of these experiments (Figure 3B), the spontaneous beat rates of the father and son cardiomyocytes were significantly lower than control cardiomyocytes: 17.5, 14.2 and 62.9 beats/min, respectively. To decipher the mechanism underlying these findings we investigated the characteristics of the key pacemaker current I_f_ in control, father and son cardiomyocytes. Notably, in agreement with the slower automaticity, I_f_ density was smaller in the mutated cardiomyocytes compared to control, as shown by the representative recordings and the I–V relations (Figure 3C,D). In addition, I_f_ density was smaller in the father than in son cardiomyocytes (Figure 3D).

#### 2.2.2. Prolonged Action Potential Duration and Increased I_Ca,L_ in LMNA-Mutated Cardiomyocytes

**Figure 4:**
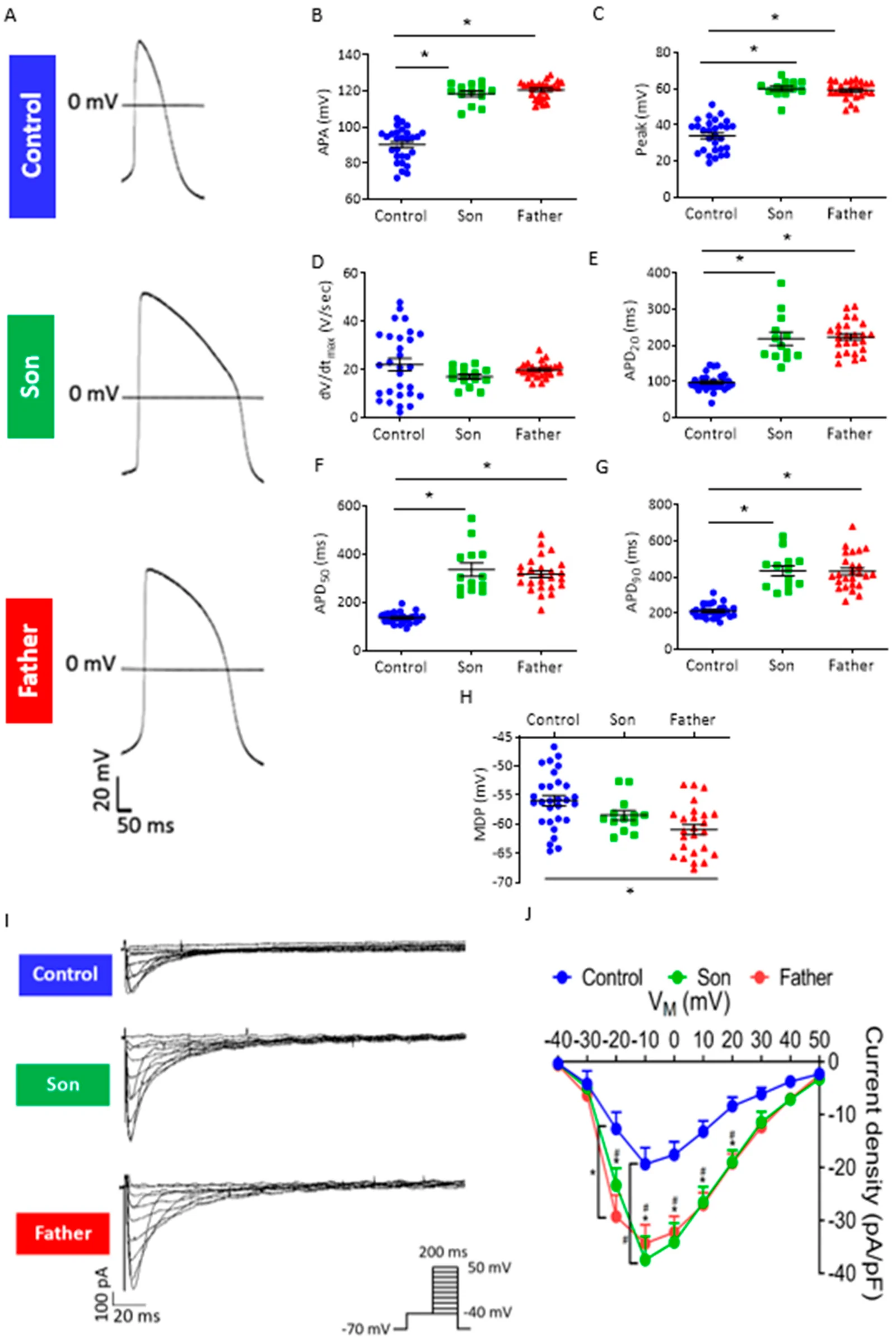
Action potential characteristics and I_Ca,L_ in control, son and father iPSC-CMs. (**A**) Representative APs of control, son and father iPSC-CMs. (**B**–**H**) Summary of AP parameters in control and *LMNA*-mutated iPSC-CMs: (**B**) APA, (**C**) AP peak, (**D**) dV/dt_max_, (**E**) APD_20_, (**F**) APD_50_, (**G**) APD_90_ and (**H**) MDP. Control (FSE-5 m and 24.5), *n* = 30; son, *n* = 14; father, *n* = 27. One-way ANOVA was performed followed by Holm–Sidak post hoc analysis. * *p* < 0.05. (**I**) Representative recordings of the I_Ca,L_ in control, son and father iPSC-CMs. (**J**) I_Ca,L_ current–voltage (I–V) relations in control and *LMNA*-mutated iPSC-CMs (*n* = 13 control (FSE-5 m); *n* = 8 son; *n* = 13 father). Two-way ANOVA was performed followed by Holm–Sidak post hoc analysis. * *p* < 0.05 father vs. control; # *p* <0.05 son vs. control.

Next, we compared key action potential (AP) characteristics in control, father and son spontaneously beating cardiomyocytes (Figure 4A–H). Whereas the maximal rate of rise of the AP upstroke (dV/dt_max_) was comparable between the three groups, both father and son cardiomyocytes showed increased AP amplitude (APA) and AP peak, as well as prolonged AP duration (APD) at 20%, 50% and 90% repolarization (APD_20_, APD_50_ and APD_90_, respectively) (Figure 4B–G). Further, the father but not son cardiomyocytes showed decreased maximum diastolic potential (MDP) compared to control cardiomyocytes (Figure 4H). As APD is rate-dependent, shortening by higher beat rates and prolonging by lower beat rates [16,17], we tested whether the prolonged APDs in the mutated cardiomyocytes were due to their decreased beat rates compared to control cardiomyocytes. Therefore, we rate-corrected the APD_90_ of *LMNA*-mutated and control cardiomyocytes by means of the following formulas used for QT interval correction: Bazett (Bazett-cAPD_90_), Fridericia (Fridericia-cAPD_90_), Framingham (Framingham-cAPD_90_) and Hodges (Hodges-cAPD_90_) [18]. Compared to control, *LMNA*-mutated cardiomyocytes showed prolonged cAPD_90_ after rate-correction using Fridericia_,_ Framingham and Hodges formulas (Supplementary Materials, Figure S1A–D), indicating that *LMNA*-mutated cardiomyocytes have inherently longer APD. To decipher the mechanism underlying APD prolongation in the father and son cardiomyocytes, we analyzed the properties of I_Ca,L_, the major inward current during the action potential plateau phase. As illustrated by the representative recordings (Figure 4I) and the I–V relations (Figure 4J), both father and son cardiomyocytes exhibited larger I_Ca,L_ density compared to control cardiomyocytes (from −20 mV to +20 mV).

### 2.3. LMNA-Mutated Cardiomyocytes Are Arrhythmogenic

**Figure 5:**
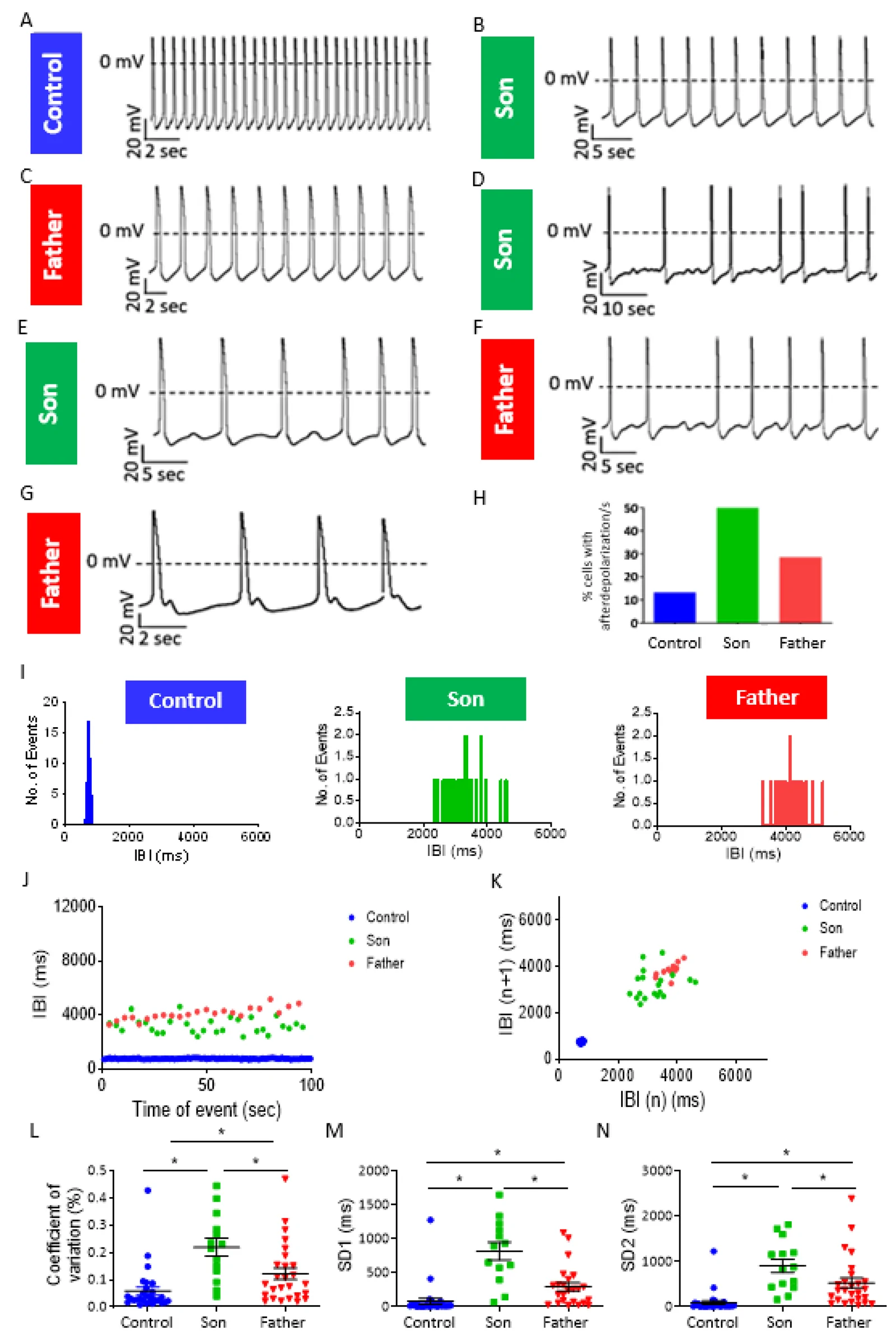
Afterdepolarizations and increased BRV in son and father *LMNA*-mutated iPSC-CMs. (**A**–**C**) Representative recordings showing afterdepolarization-free spontaneous activity in control, son and father cardiomyocytes. (**D**,**E**) Spontaneous AP recordings demonstrating DADs in son iPSC-CMs. (**F**,**G**) Spontaneous AP recordings demonstrating afterdepolarizations in father iPSC-CMs. (**H**) Percentage of experiments with afterdepolarization/s in control, son and father cardiomyocytes (*n* = 30 control (FSE-5 m and 24.5); *n* = 14 son; *n* = 28 father). (**I**) Representative IBI histograms in control, son and father cardiomyocytes. (**J**) Superimposed representative IBI vs. time plots of control, son and father iPSC-CMs. (**K**) Superimposed representative Poincaré plots of control, son and father iPSC-CMs. (**L**) COV analysis in control, father and son cardiomyocytes. (**M**) SD1 analysis in control, father and son cardiomyocytes. (**N**) SD2 analysis in control, father and son cardiomyocytes. Control (FSE-5 m and 24.5), *n* = 30; son, *n* = 14; father, *n* = 27. One-way ANOVA was performed followed by Holm–Sidak post hoc analysis. * *p* < 0.05.

Next, we investigated whether *LMNA*-mutated cardiomyocytes presented electrophysiological abnormalities which may underlie the clinical lethal arrhythmias. Although *LMNA*-mutated iPSC-CMs display areas of regular beating patterns (like control), 50% and 29% of the son and father cardiomyocytes, respectively, exhibited afterdepolarization event(s) (Figure 5A–H). These abnormal depolarizations were mainly of the delayed afterdepolarizations (DAD)-type; (7/14 and 7/28 of experiments in son and father cardiomyocytes, respectively) (Figure 5D–F), and rarely of the early afterdepolarization (EAD)-type (1/14 and 1/28 of experiments in son and father cardiomyocytes, respectively) (Figure 5G). To quantify the arrhythmogenic firing patterns of *LMNA*-mutated cardiomyocytes, we analyzed their Beat Rate Variability (BRV) representing the dynamic non-linear features of automaticity. As illustrated by the representative Inter-Beat-Interval (IBI) histograms, IBI vs. time plots and Poincaré plot clouds (in which each IBI is plotted against the preceding IBI), *LMNA*-mutated cardiomyocytes displayed an increased range of IBIs compared to control cardiomyocytes (Figure 5I–K). Additionally, compared to control cardiomyocytes, mutated cardiomyocytes exhibited an increased coefficient of variation (COV) and larger standard descriptors SD1 and SD2 representing short-term variability and long-term variability, respectively (Figure 5L–N). Further, the son’s COV, SD1 and SD2 were larger than the father’s cardiomyocytes. In support of the patch-clamp findings, recordings of extracellular electrograms from spontaneously beating clusters, using the MEA data acquisition system, showed that 100% of the father and 83% of the son clusters presented arrhythmias, compared to 20% in control (Supplementary Materials, Figure S2A–G).

#### 2.3.1. Abnormal Response to β-Adrenergic Stimulation in LMNA-Mutated iPSC-CMs

**Figure 6:**
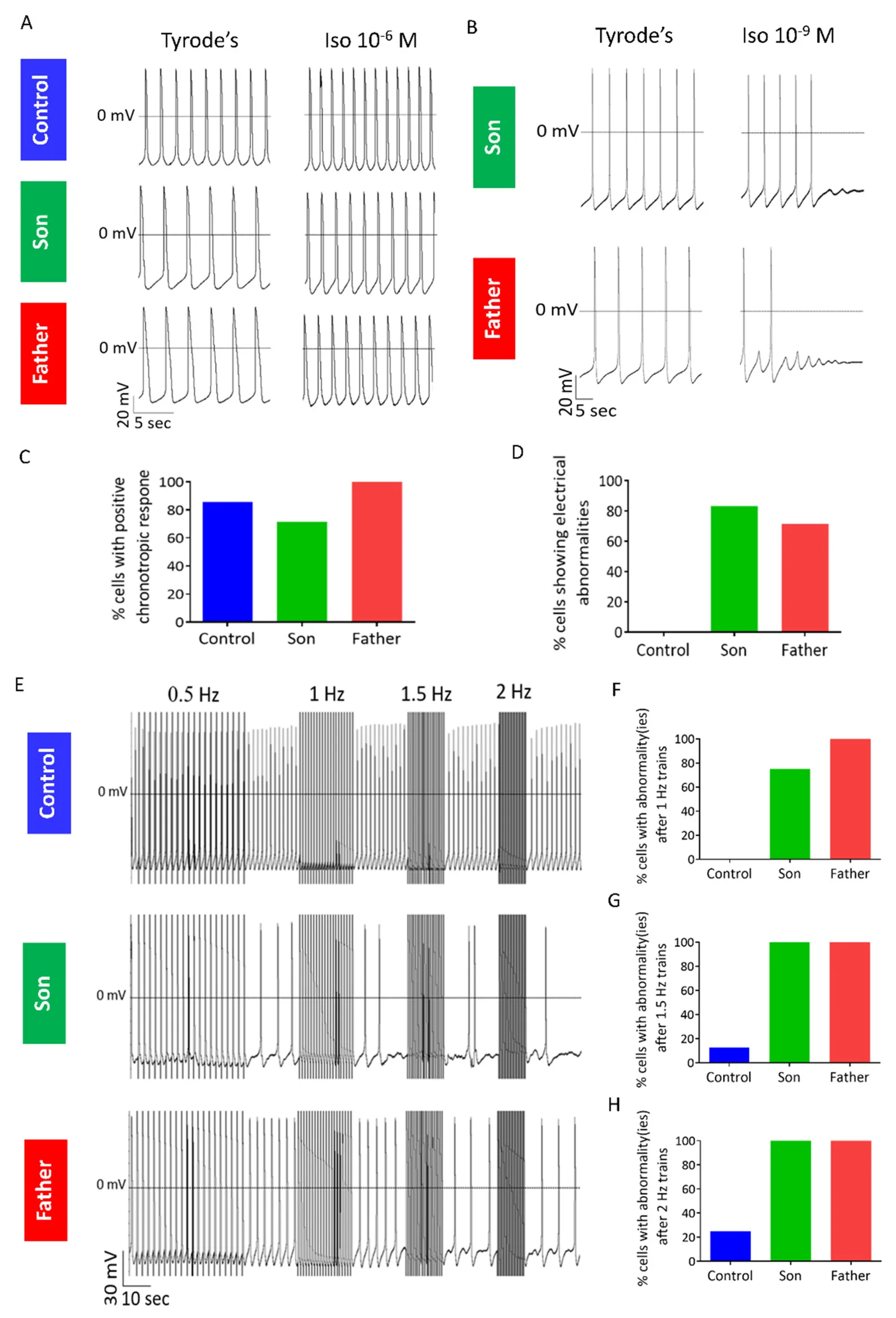
Abnormal response of *LMNA*-mutated cardiomyocytes to β-adrenergic stimulation and rapid pacing. (**A**) AP recordings from control, son and father cardiomyocytes showing a positive chronotropic response to isoproterenol. (**B**) Representative AP recordings from father and son cardiomyocytes showing afterdepolarization/s and cessation of spontaneous AP firing in response to isoproterenol. (**C**) Percentage of cells showing positive chronotropic response to isoproterenol (*n* = 7 control; *n* = 7 son; *n* = 7 father). (**D**) Percentage of cells showing electrophysiological abnormalities in response to isoproterenol (*n* = 7 control; *n* = 6 son; *n* = 7 father). (**E**) Representative recordings of trains protocol in control, son and father cardiomyocytes. (**F**–**H**) Percentage of cells showing electrical abnormality(ies) following stimulation at (**F**) 1 Hz (*n* = 6 control (FSE-5 m and 24.5); *n* = 4 son; *n* = 4 father), (**G**) 1.5 Hz (*n* = 8 control (FSE-5 m and 24.5); *n* = 4 son; *n* = 4 father) and (**H**) 2 Hz (*n* = 8 control (FSE-5 m and 24.5); *n* = 4 son; *n* = 3 father).

The β-adrenergic signaling pathway has a prominent role in regulating normal cardiac function, but is also involved in the pathogenesis of heart failure [19,20]. In addition, several studies have shown exacerbation of phenotype in response to β-adrenergic stimulation in iPSC-CMs modelling different cardiac diseases such as hypertrophic cardiomyopathy, long QT syndrome type 2, and catecholaminergic polymorphic ventricular tachycardia (CPVT) [21,22,23]. At the cellular level, β-adrenergic stimulation leads to increased Ca^2+^ influx (via the L-type Ca^2+^ channel (LTCC) and increased Ca^2+^ uptake into the sarcoplasmic reticulum (SR), leading to enhanced SR Ca^2+^ load [24]. Therefore, to determine whether this pathway is involved in *LMNA*-cardiomyopathy, we investigated the response of *LMNA*-mutated cardiomyocytes to β-adrenergic stimulation by exposure to isoproterenol (10^−9^–10^−6^ M). As expected, in the majority of control (85.7%), son (71.4%) and father (100%) cardiomyocytes, isoproterenol caused a positive chronotropic response (at least in one of the concentrations), demonstrating the functionality of the β-adrenergic signaling cascade in the three groups (Figure 6A,C). However, in 5/6 of son cardiomyocytes and 5/7 of father cardiomyocytes, but in none of the control cardiomyocytes, isoproterenol-induced DADs and/or cessation of spontaneous firing (Figure 5B,D).

#### 2.3.2. Abnormal Response to Rapid Pacing in *LMNA*-Mutated iPSC-CMs

To determine whether the β-adrenergic agonist-induced electrophysiological abnormalities in *LMNA*-mutated cardiomyocytes were specific to stimulation of this pathway or rather resulted from the downstream effect of elevated [Ca^2+^]_i_, we investigated the response of *LMNA*-mutated iPSC-CMs to rapid pacing, a condition causing diastolic [Ca^2+^]_i_ elevation [25]. Specifically, we applied trains protocol during which cardiomyocytes were electrically paced at increasing frequencies, each followed by a 20-s pause. Notably, compared to control, an increased percentage of father and son cardiomyocytes showed electrical abnormalities such as DADs, arrhythmias or cessation of firing during the post-pacing intervals (Figure 6E–H). These results demonstrate the abnormal function of *LMNA*-mutated cardiomyocytes following yet another condition causing elevated [Ca^2+^]_i_.

#### 2.3.3. Abnormal Caffeine-Induced Ca^2+^ Release in LMNA-Mutated Cardiomyocytes

The generation of DADs in *LMNA*-mutated cardiomyocytes point to increased cytosolic Ca^2+^ levels ([Ca^2+^]_i_), which is consistent with the increased susceptibility of the mutated cardiomyocytes to interventions causing elevated [Ca^2+^]_i_. In turn, increased [Ca^2+^]_i_ can result, for example, from leaky Ryanodine receptor (RyR), which will lead to decreased sarcoplasmic reticulum (SR) Ca^2+^ content. To investigate this option, we measured the SR Ca^2+^ release upon application of 10 mM caffeine. Compared to control, the father *LMNA*-mutated cardiomyocytes showed altered response to caffeine (10 mM) characterized by a smaller change in Ca^2+^ signal amplitude and area, and a shorter recovery time (Supplementary Materials, Figure S3A–D). These results suggest that decreased SR Ca^2+^ content in *LMNA*-mutated cardiomyocytes may contribute to the increased cytosolic [Ca^2+^]_i_.

#### 2.3.4. KB-R7943 Eliminates DADs in LMNA-Mutated Cardiomyocytes

**Figure 7:**
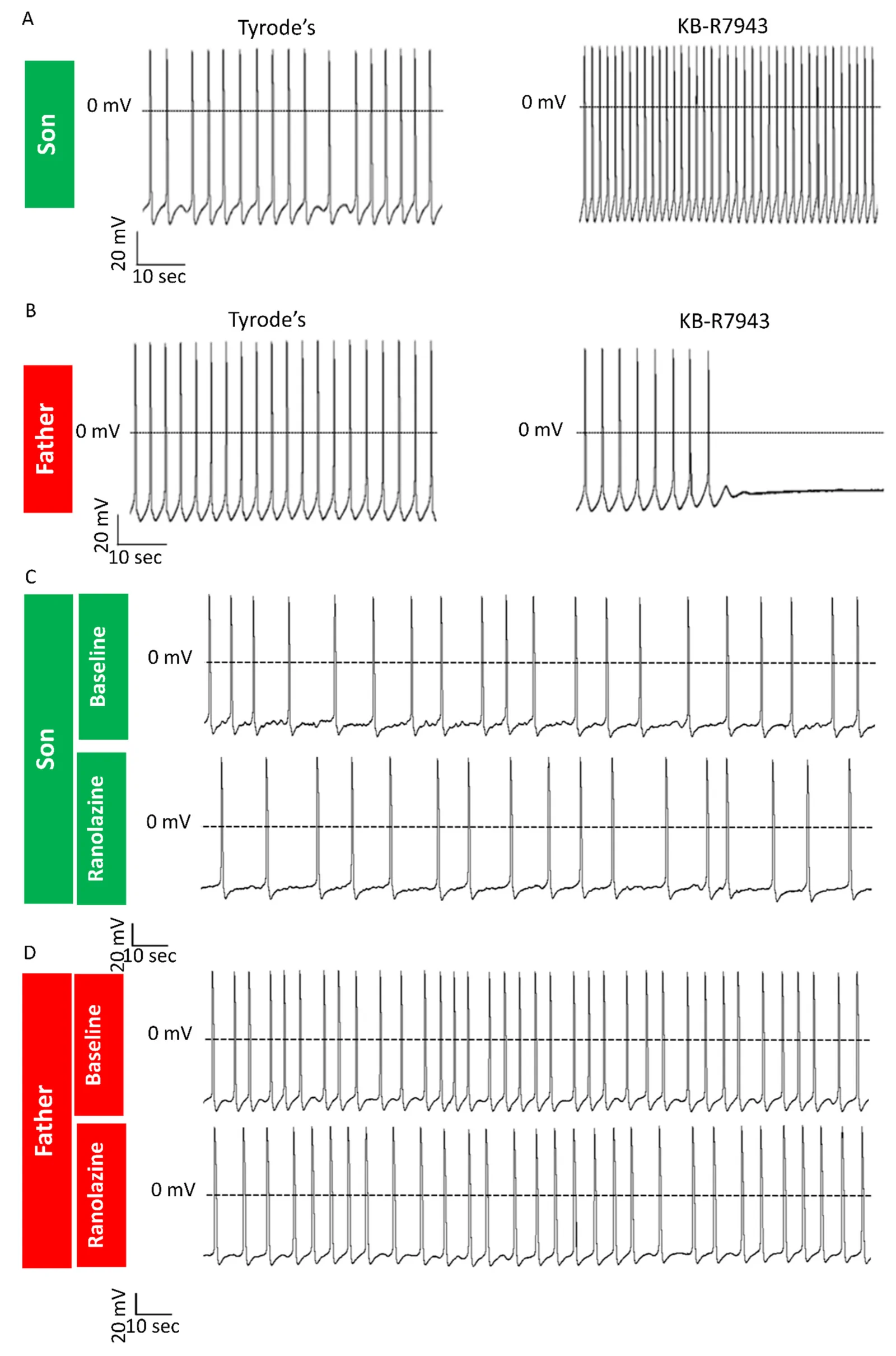
Effects of KB-R7943 and ranolazine in *LMNA*-mutated cardiomyocytes. AP recordings demonstrating different effects of KB-R7943: (**A**) Elimination of DADs in the son *LMNA*-mutated cardiomyocytes; (**B**) Cessation of spontaneous firing in the father *LMNA*-mutated cardiomyocytes showing no DADs. AP recordings from the son (**C**) and father (**D**) cardiomyocytes showing afterdepolarizations and arrhythmias before and after application of ranolazine (10 μM).

The Na^+^/Ca^2+^ exchanger (NCX) is a bidirectional electrogenic ion transporter that exchanges one Ca^2+^ ion for three Na^+^ ions across the plasma membrane of cardiomyocytes [26]. In addition to its physiological role in Ca^2+^ homeostasis, NCX was shown to be involved in the pathogenesis of different cardiac conditions such as arrhythmias, ischemia and heart failure [27]. As DADs are known to result from elevated [Ca^2+^]_i_, we tested whether inhibition of the reverse-mode of NCX, which imports Ca^2+^ into the cell, will alleviate Ca^2+^ overload conditions and abolish DADs in *LMNA*-mutated iPSC-CMs. Notably, the application of KB-R7943 (3 μM) eliminated the DADs in *LMNA*-mutated cardiomyocytes (son, *n* = 3/3) (Figure 7A). Since (as described above) not all mutated cardiomyocytes present DADs and arrhythmias, we also tested the effect of KB-R7943 on *LMNA*-mutated cardiomyocytes showing no DADs. For a yet unexplained reason, application of KB-R7943 caused cessation of spontaneous beating in this subgroup of *LMNA*-mutated cardiomyocytes (father, *n* = 3/4) (Figure 7B). Overall, these results suggest a specific beneficial effect of KB-R7943 on *LMNA*-mutated cardiomyocytes with DADs but not on those without DADs.

#### 2.3.5. Investigating the Anti-Arrhythmic Efficacy of Ranolazine

The late sodium current (I_Na,L_) inhibitor ranolazine was shown to suppress EADs, DADs and triggered activity in mouse *LMNA*^N195K/N195K^ ventricular myocytes [28]. Therefore, we tested whether ranolazine exerts similar anti-arrhythmic effects in a human model of *LMNA*-mutated cardiomyocytes. Because ranolazine did not eliminate DADs and arrhythmias in *LMNA*-mutated cardiomyocytes (Figure 7C,D) (son *n* = 6/6, father *n* = 3/3), we concluded that arrhythmias in *LMNA*-mutated cardiomyocytes are not due to increased I_Na,L_.

### 2.4. Structural and Gene Expression Models in LMNA-Mutated iPSC-CMs

Two main hypotheses were proposed to explain how *LMNA* mutations result in different diseases: (1) the structural hypothesis—proposing that *LMNA* mutations result in nuclear integrity defects and cellular damage in tissues subjected to mechanical stress, such as cardiac and skeletal muscles. (2) The gene expression hypothesis—proposing alterations in gene expression patterns in the affected cells [29,30]. Accordingly, we investigated whether *LMNA*-mutated iPSC-CMs display nuclear morphological abnormalities and/or gene expression alterations.

#### 2.4.1. Ultrastructural Changes in LMNA-Mutated iPSC-CMs

**Figure 8:**
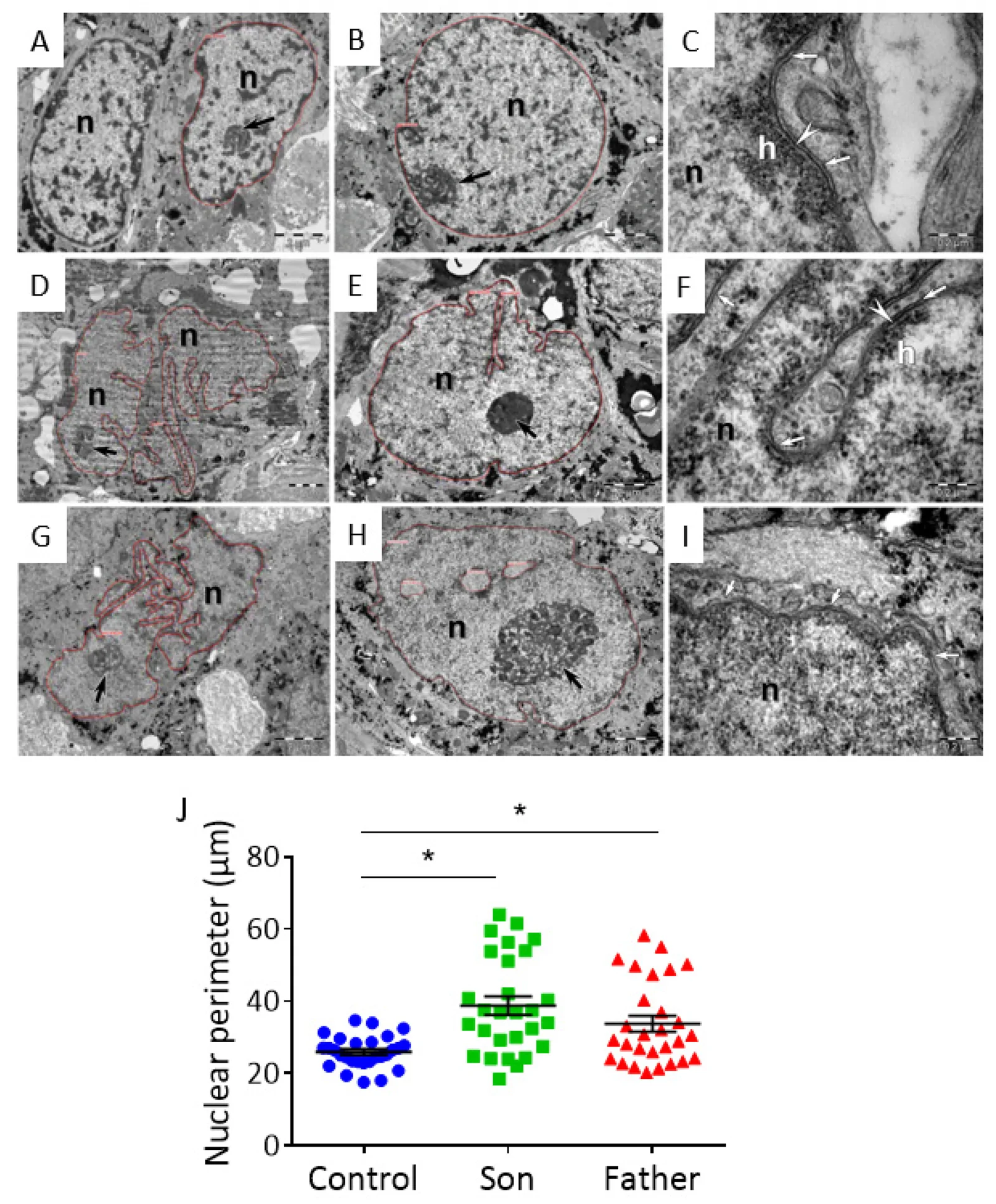
TEM images of *LMNA*-mutated and control iPSC-CMs. TEM images from control (FSE-5 m) (**A**–**C**), father (**D**–**F**) and son (**G**–**I**) iPSC-CMs. Thin red lines mark nuclear silhouettes. Black arrows point out the nucleoli. (**A**,**B**) The nuclei (n) of control iPSC-CMs show a smooth contour and scarce heterochromatin, mostly associated with the nuclear envelope (NE). Heterochromatin clumps are also visible inside the nucleus. (**B**) The nucleolus (arrow) with heterogeneous appearance is associated with peripheral heterochromatin. (**C**) Higher magnification shows nuclear lamina (NL) (arrowhead) attaching the heterochromatin (h) to the NE (arrows). (**D**,**E**) The nuclei (n) of father iPSC-CMs show an irregular contour and indentations. The heterochromatin is limited to the nuclear periphery. (**E**) The nucleolus (arrow) is densely packed. (**F**) Higher magnification shows short segments of NL (arrowhead). The heterochromatin (h) is scarcely attached to the NE (arrows). (**G**,**H**) The nuclei (n) of son iPSC-CMs show highly indented nuclear contour. (**H**) The heterochromatin is almost absent in the nucleus and the nucleolus (arrow) is large with numerous visible fibrillary centers. (**I**) Higher magnification shows the membranes of the NE (arrows). There is no heterochromatin attached and the lamina is inconspicuous. (**J**) Comparison of nuclear perimeter in control, father and son iPSC-CMs (*n* = 29 for each group). One-way ANOVA was performed followed by Holm–Sidak post hoc analysis. * *p* < 0.05. Scale bar represents: in (**A**,**B**,**D**,**E**,**G**,**H**)—2.0 µm, in (**C**,**F**,**I**)—0.2 µm.

To determine whether the E342K *LMNA* mutation is associated with nuclear structural irregularities, we investigated the nuclear morphology of control and *LMNA*-mutated iPSC-CMs. Transmission Electron Microscopy (TEM) analysis showed that control iPSC-CMs have mostly euchromatic nuclei with clumps of heterochromatin attached to the nuclear envelope (NE) or dispersed inside the nuclei (Figure 8A–C). The nuclear lamina (NL) (~25 nm wide) was clearly visible where the NE was sectioned transversally (Figure 8C). In contrast to control specimens, the father and son *LMNA*-mutated iPSC-CMs showed highly indented nuclei and sparse (Figure 8D–F) or almost absent heterochromatin (Figure 8G,H). Particularly, chromatin was highly dispersed in nuclei of son iPSC-CMs (Figure 8G,H), and the NL was invisible (Figure 8I). In addition, both father and son iPSC-CMs showed significantly increased nuclear perimeter compared to control iPSC-CMs (Figure 8J).

#### 2.4.2. Gene Expression Alterations in LMNA-Mutated iPSC-CMs

**Figure 9:**
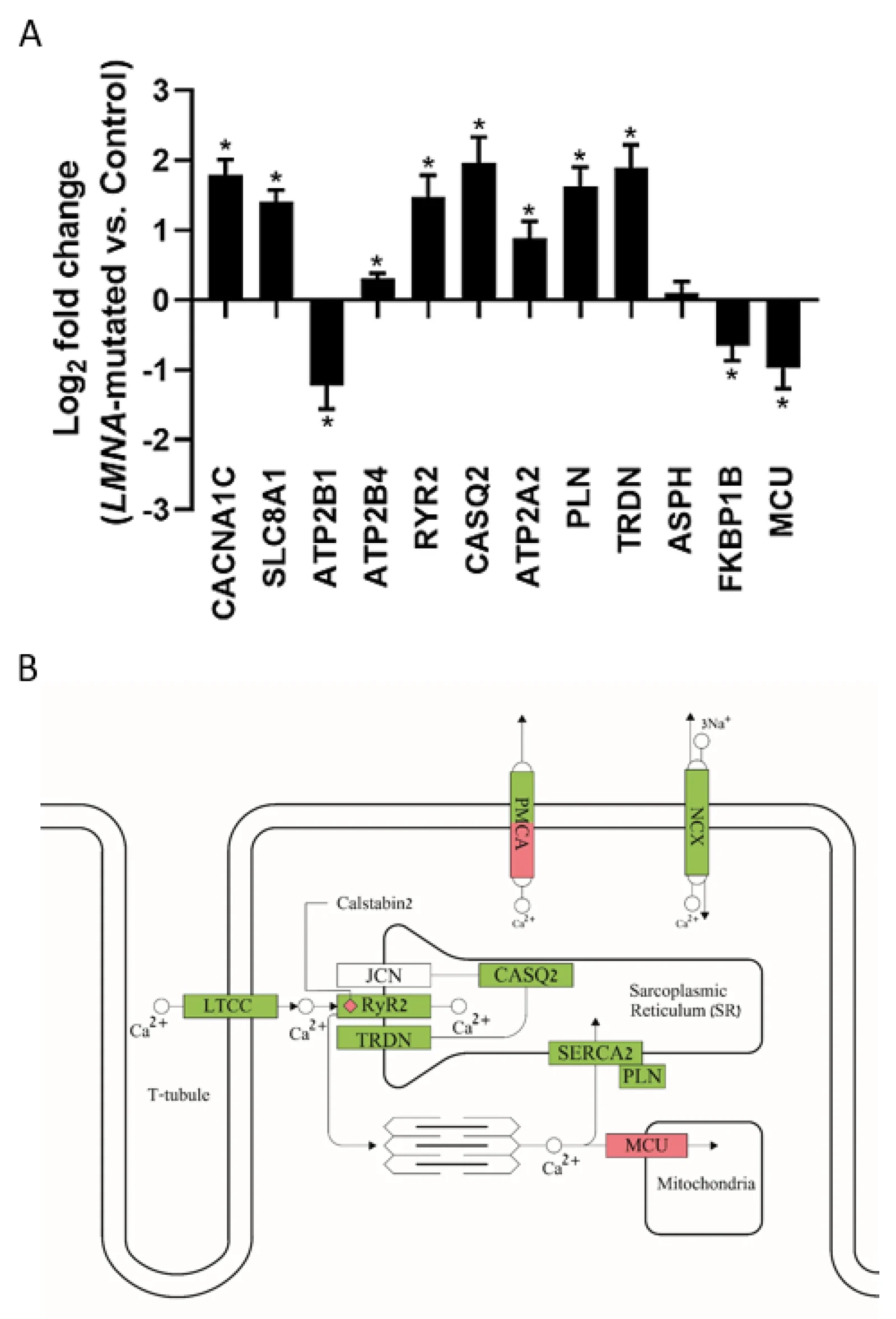
Altered gene expression in *LMNA*-mutated iPSC-CMs. (**A**) Expression of genes involved in Ca^2+^ handling in *LMNA*-mutated iPSC-CMs compared to control. * padj < 0.05 *LMNA*-mutated vs. control. (**B**) Schematic illustration of a *LMNA*-mutated cardiomyocyte showing the expected effect of its altered gene expression on key Ca^2+^ handling proteins: LTCC, plasma membrane Ca^2+^ ATPase (PMCA), NCX, RyR2, calsequestrin 2 (CASQ2), triadin (TRDN), junctin (JCN), calstabin2, SERCA2, phospholamban (PLN) and mitochondrial Ca^2+^ uniporter (MCU). Green indicates upregulation; Red indicates downregulation. White indicates gene expression alteration which is not statistically significant.

To investigate whether *LMNA*-related DCM leads to altered gene expression, we performed RNA-seq analysis in control and in father and son *LMNA*-mutated iPSC-CMs. Both principal component analysis (PCA) and heatmap revealed that the two different clones of control iPSC-CMs (FSE-5 m and 24.5) clustered close to each other and far from the father iPSC-CMs, with the son iPSC-CMs clustering in between (Supplementary Materials, Figure S4A,B). As shown in the volcano plot (Supplementary Materials, Figure S4C), *LMNA*-mutated iPSC-CMs showed a total of 9794 differentially expressed genes (DEGs) compared to control iPSC-CMs. Ingenuity Pathway Analysis (IPA) revealed the top 20 canonical pathways associated with DEGs, including: mitochondrial dysfunction, cardiac hypertrophy signaling and calcium signaling (Supplementary Materials, Figure S4D). As our aforementioned results pointed to Ca^2+^ handling abnormalities in *LMNA*-mutated cardiomyocytes, we investigated the expression of genes encoding key proteins involved in the Ca^2+^ cycling of cardiomyocytes (Figure 9A). In accordance with our findings of increased I_Ca,L_ density in both father and son cardiomyocytes, *LMNA*-mutated iPSC-CMs showed increased expression of the *CACNA1C* gene, encoding the Ca^2+^ voltage-gated channel subunit alpha1 C. Additionally, *LMNA*-mutated iPSC-CMs showed increased expression of the *SLC8A1* gene encoding the NCX1, a prominent contributor to the generation of the transient inward current (I_ti_) responsible for DAD formation. Furthermore, *LMNA*-mutated iPSC-CMs showed increased expression of different genes encoding for major proteins involved in the SR Ca^2+^ cycling such as *RYR2* (encoding ryanodine receptor 2), *CASQ2* (encoding calsequestrin 2), *ATP2A2* (encoding SERCA2), *PLN* (encoding phospholamban) and *TRDN* (encoding triadin). In addition, *LMNA*-mutated iPSC-CMs showed decreased expression of *FKBP1B*, encoding for calstabin2 which stabilizes the closed state of the RyR2. Figure 9B shows a schematic illustration of an *LMNA*-mutated cardiomyocyte with the expected effects of its altered gene expression (of the genes shown in Figure 9A) on key Ca^2+^ handling proteins. Overall, these findings demonstrate major alterations in the gene expression profile, including in genes encoding key Ca^2+^ handling proteins, in *LMNA*-mutated cardiomyocytes.

## 3. Discussion

Our main findings are that *LMNA*-mutated iPSC-CMs exhibit the following abnormalities compared to control: (1) decreased spontaneous beat rate and I_f_ density; (2) prolonged APD and higher I_Ca,L_ density; (3) DADs and arrhythmias; (4) electrical abnormalities in response to interventions causing elevated [Ca^2+^]_i_; (5) nuclear morphological abnormalities; (6) gene expression alterations, including in genes encoding for major Ca^2+^ handling proteins. In addition, the application of KB-R7943 abolished the DADs in *LMNA*-mutated iPSC-CMs.

### 3.1. Electrophysiological Abnormalities in LMNA-Mutated Cardiomyocytes

#### 3.1.1. Decreased Spontaneous Beat Rate and I_f_ Density

The father and son *LMNA*-mutated cardiomyocytes showed decreased spontaneous beat rate; this finding is of high importance as *LMNA*-related DCM can manifest with sinus bradycardia [10]. This finding in cardiomyocytes devoid of autonomic innervation, implies the involvement of an intrinsic pacemaking mechanism. Indeed, we found decreased I_f_ density, a key determinant of the diastolic depolarization [31], in *LMNA*-mutated cardiomyocytes compared to control. In this regard, it should be noted that mutations in the *HCN4* gene encoding the HCN4 channels that conduct I_f_, were reported in patients with sinus bradycardia [32,33]. In addition to their prominent role in SA node automaticity, the I_f_ channels are also expressed in the AV node [34]. Accordingly, induction of HCN4 knockout in a mouse model resulting in decreased I_f_ led to both bradycardia as well as AV-block [35]. Therefore, further study is needed to investigate whether decreased I_f_ is involved in the generation of AV-block in *LMNA*-related DCM.

#### 3.1.2. Altered Action Potential Parameters and Increased I_Ca,L_ Density

The main change in action potential characteristics in *LMNA*-mutated cardiomyocytes was prolonged APD, which on the whole-body level, may correlate with increased QT interval leading to life-threatening ventricular arrhythmias. In this regard, prolonged QT interval was described in several HGPS patients carrying an *LMNA* mutation [36]. Yet, further research is needed in order to evaluate whether QT prolongation is involved in arrhythmias observed in *LMNA*-related DCM. Mechanistically, we found that both father and son *LMNA*-mutated cardiomyocytes showed increased I_Ca,L_ density, the main inward current constituting the AP plateau phase [37]. Of note, a recent study in *LMNA*-mutated iPSC-CMs reported on the upregulation of CACNA1C encoding the pore-forming subunit of LTCC [38].

#### 3.1.3. Arrhythmias and DADs

As illustrated by the action potential recordings and extracellular electrograms, the spontaneously firing patterns of father and son cardiomyocytes were highly arrhythmogenic. Specifically, *LMNA*-mutated cardiomyocytes showed prominent DADs, arrhythmias and increased BRV. Mechanistically, the occurrence of DADs points to [Ca^2+^]_i_-overload in *LMNA*-mutated cardiomyocytes. Noteworthy and in support of our results, DADs and arrhythmias were reported recently in *LMNA*-mutated iPSC-CMs [39].

#### 3.1.4. Altered Response to β-Adrenergic Stimulation, Rapid Pacing and Caffeine Application in LMNA-Mutated Cardiomyocytes

In *LMNA*-mutated cardiomyocytes, β-adrenergic stimulation, as well as rapid pacing, resulted in electrical abnormalities (e.g., DADs and cessation of AP firing), suggesting the involvement of a downstream common factor—[Ca^2+^]_i_ elevation. Therefore, we propose the following mechanism: *LMNA*-mutated cardiomyocytes display elevated [Ca^2+^]_i_ under baseline condition, accounting for their arrhythmogenic firing patterns. When *LMNA*-mutated cardiomyocytes are exposed to β-adrenergic stimulation or rapid pacing, [Ca^2+^]_i_ is further elevated, causing arrhythmias and/or cessation of spontaneous firing. Our findings of decreased (compared to control) Ca^2+^ signal amplitude and area following caffeine application, suggest that due to the “leaky” RyR the SR Ca^2+^ stores are depleted, thus causing elevated cytosolic [Ca^2+^]_i_ in *LMNA*-mutated cardiomyocytes.

#### 3.1.5. KB-R7943 Eliminates DADs in LMNA-Mutated Cardiomyocytes

We found that the application of the reverse-mode NCX inhibitor, KB-R7943, resulted in the elimination of DADs in *LMNA*-mutated cardiomyocytes. This may be due to decreased Ca^2+^ influx and thereby alleviation of the [Ca^2+^]_i_ overload, leading consequently to decreased transient inward current (I_ti_) which is primarily conducted by the forward mode of NCX and is involved in the generation of DADs [40].

#### 3.1.6. Ranolazine Does Not Eliminate DADs and Arrhythmias in LMNA-Mutated Cardiomyocytes

Increased I_Na,L_ leads to elevated [Na^+^]_i_ which in turn may result in increased [Ca^2+^]_i_ (through the reverse-mode NCX), possibly culminating in DADs [41]. However, since ranolazine did not block arrhythmias in both father and son cardiomyocytes, it is unlikely that increased I_Na,L_ is the cause of arrhythmias in the *LMNA*-mutated cardiomyocytes. On the broader scale, our findings from this section demonstrate our ability to test different drugs using *LMNA*-mutated iPSC-CMs in order to: (1) elucidate cellular mechanisms involved in the disease; (2) search for beneficial pharmacological interventions.

### 3.2. The Probable Association between LMNA Mutations and Electrophysiological Abnormalities

Two main hypotheses were proposed to explain how *LMNA* mutations result in different tissue-specific pathologies: (1) the structural hypothesis and (2) the gene expression hypothesis.

#### 3.2.1. Ultrastructural Abnormalities in LMNA-Mutated iPSC-CMs

The TEM analysis revealed several typical abnormalities in the nuclei of *LMNA*-mutated iPSC-CMs. First, we found altered morphology with indentations and increased nuclear perimeter compared to control iPSC-CMs. Additionally, the heterochromatin of *LMNA*-mutated iPSC-CMs appeared missing or scant compared to control. As heterochromatin is generally transcriptionally silent [42], such aberrations may imply changes in the gene expression of *LMNA*-mutated iPSC-CMs. In this regard, it should be noted that loss of peripheral heterochromatin was reported in the nuclei of HGPS cells with *LMNA* mutation [43]. Overall, our results suggest that the E342K *LMNA* mutation is associated with nuclear structural abnormalities such as altered morphology and heterochromatin disorganization.

#### 3.2.2. Gene Expression Alterations in LMNA-Mutated iPSC-CMs

*LMNA*-mutated iPSC-CMs showed extensive alterations in their gene expression profile compared to control iPSC-CMs, with a total of 9794 DEGs. These findings are in line with the gene expression hypothesis suggesting that *LMNA* mutations lead to gene expression alterations in the mutated cells. Specifically, we focused on *LMMA*-mutated iPSC-CMs changes in the expression of genes encoding major proteins involved in the Ca^2+^ handling machinery. These alterations can explain key electrophysiological findings in *LMNA* iPSC-CMs: (1) increased I_Ca,L_ density and consequently elevated [Ca^2+^]_i_ levels; (2) DADs and arrhythmias, probably due to increased I_ti_ mediated by the NCX. Additionally, a smaller (compared to control) change in Ca^2+^ signal in response to caffeine application can be explained by RyR2 Ca^2+^ leak ascribed to decreased levels of calstabin2 in the context of elevated levels of other key proteins involved in the SR Ca^2+^ cycling (such as RyR2, calsequestrin 2, SERCA2, phospholamban and triadin). Collectively, these findings suggest gene expression alterations as a possible underlying mechanism leading to electrophysiological abnormalities in *LMNA*-mutated iPSC-CMs.

### 3.3. Proposed Mechanism of Arrhythmias in LMNA-Mutated iPSC-CMs

Our findings suggest an integrated model of both structural and gene expression alterations leading to electrophysiological abnormalities and arrhythmias in *LMNA*-mutated cardiomyocytes; *LMNA* mutation results in nuclear morphological abnormalities including alterations in nuclear shape and perimeter size as well as in heterochromatin organization. These irregularities lead to broad changes in the gene expression profile of the mutated cells. Among these are alterations in the expression of genes encoding for prominent proteins involved in the Ca^2+^ handling machinery of the diseased cardiomyocytes. Eventually, these changes result in elevated [Ca^2+^]_i_ levels and consequently in DADs and arrhythmias.

## 4. Materials and Methods

### 4.1. LMNA-Mutated Dermal Fibroblasts

Dermal biopsies were obtained from a 63-year-old male DCM patient and his 35-year-old son, both carrying the E342K missense mutation in the LMNA gene, leading to the substitution of nucleotide G to A, thus exchanging the negatively charged glutamic acid to the positively charged lysine. The donors signed a consent form according to approval #3116 by the Helsinki Committee for experiments on human subjects at Rambam Health Care Campus, Haifa, Israel. Informed consent was obtained before all donations.

### 4.2. Generation of LMNA-Mutated iPSCs

For *LMNA*-mutated iPSCs generation, father and son human dermal fibroblasts were infected in two cycles with a single polycistronic lentiviral vector harboring the STEMCCA cassette, which contains the 4 transcription factors: Oct4, Sox2, Klf4 and c-Myc, as previously described [44]. Clones 16.5 and 21.10 were selected from father and son iPSCs, respectively. As control, we used the two following clones: (i) clone FSE-5 m (generated from neonatal foreskin fibroblasts) as previously described [45]; (ii) clone 24.5 (generated from a 42-year old female) as previously described [46].

### 4.3. Karyotype Analysis

Karyotype analysis was performed using standard G-banding chromosome analysis by the cytogenetic laboratory according to standard procedures [23].

### 4.4. Genotyping

PCR reaction was performed on the genomic DNA with primers that delimit the mutation area on the *LMNA* gene. Genomic DNA was purified using the QIAGEN DNeasy Blood and Tissue Kit (Hilden, Germany). PCR was performed to the *LMNA* gene using the primers: F-5′-GGGAGCTCACCAAACCCT-3′ and R-5′-AGAGGACACTGCCAGCACCT-3′. Following PCR, we performed sequencing of the genomic PCR product.

### 4.5. Immunofluorescence Staining

iPSC colonies were grown for 3 days on Matrigel™-coated coverslips suitable for immunofluorescence staining. The cells were fixed in 4% paraformaldehyde for 10 min at room temperature, rinsed twice with phosphate-buffered saline (PBS) and permeabilized in 0.5% Triton X-100 diluted in PBS for 10 min. Samples were then blocked with 2% FBS in PBS for 10 min at room temperature. The primary antibodies added were for typical pluripotency markers rabbit anti-Oct3/4 (1:100; Santa Cruz, CA, USA), goat anti-Nanog (1:20; R&D, Minneapolis, MN, USA), mouse anti-Sox2 (1:100; Millipore, Santa Cruz, CA, USA), mouse anti-TRA 1–60 (1:100; Millipore), mouse anti-TRA 1–81 (1:100; Millipore), mouse anti-SSEA4 (1:100; Hybridoma Bank, Iowa City, IA, USA) and kept at room temperature for 1 h. The secondary antibodies were as follows: donkey anti-rabbit Cye 3 (1:100; Chemicon) and donkey anti-mouse/goat Alexafluor 488 (1:100; Invitrogen, Carlsbad, CA, USA). Cells were also stained with DAPI (1:1000; Boehringer, Mannheim, Germany) for nuclei detection, diluted in blocking solution, and added in darkness at room temperature for 1 h. To stain the nucleus, 0.15% DAPI was added. The coverslips with the stained preparations were then mounted on glass. The staining was visualized using laser scanning confocal inverted microscope (Axio Observer.z1, Zeiss, Oberkochen, Germany).

### 4.6. Teratomas Formation

iPSC colonies from one 6-well plate were detached using 1 mg/mL type IV collagenase, washed three times in PBS and then injected into thigh muscle of severe combined immunodeficient (SCID) mice. Teratomas were observed 8–12 weeks after injection. Tumors were excised, fixed, embedded in paraffin, sectioned and stained with hematoxylin/eosin.

### 4.7. Exome Sequencing

Genomic DNA extracted from father (clone 16.5) and son (clone 21.10) *LMNA*-mutated iPSC was studied by exome sequencing at the Illumina NextSeq platform using the Nextera-Library-Prep-Kit (Illumina, San Diego, CA, USA) and the Nextera-xGen-Exome-Research-Panel (IDT). The results were filtered for known cardiomyopathy and arrhythmia genes (*ABCC9*, *ACTC1*, *ACTN2*, *AKAP9*, *ANK2*, *ANKRD1*, *BAG3*, *CACNA1C*, *CACNA2D1*, *CACNB2*, *CALM1*, *CALR3*, *CASQ2*, *CAV3*, *CRYAB*, *CSRP3*, *CTNNA3*, *DES*, *DMD*, *DSC2*, *DSG2*, *DSP*, *DTNA*, *EMD*, *EYA4*, *FHL1*, *FKTN*, *FLNC*, *GATAD1*, *GLA*, *GNAI2*, *GPD1L*, *HCN4*, *JPH2*, *JUP*, *KCND3*, *KCNE1*, *KCNE2*, *KCNE3*, *KCNH2*, *KCNJ2*, *KCNJ5*, *KCNJ8*, *KCNQ1*, *LAMA4*, *LAMP2*, *LDB3*, *LMNA*, *MYBPC3*, *MYH6*, *MYH7*, *MYL2*, *MYL3*, *MYLK2*, *MYOZ2*, *MYPN*, *NEBL*, *NEXN*, *PDLIM3*, *PKP2*, *PLN*, *PRKAG2*, *RAF1*, *RBM20*, *RYR2*, *SCN10A*, *SCN1B*, *SCN3B*, *SCN4B*, *SCN5A*, *SDHA*, *SGCD*, *SNTA1*, *TAZ*, *TCAP*, *TGFB3*, *TMEM43*, *TMPO*, *TNNC1*, *TNNI3*, *TNNT2*, *TPM1*, *TRDN*, *TRPM4*, *TTN*, *TTR*, *VCL*) and variants were called using GensearchNGS 1.6.77 for their minor allele frequency (MAF) < 0.01 and variant specification. About 98.5% of the coding sequence including flanking intronic sequences (±10 bp) was covered at least ten times.

### 4.8. Culturing and Differentiation of iPSCs into Cardiomyocytes

iPSCs were cultured on Matrigel (GFR, BD Biosciences, Franklin Lakes, NJ, USA) coated plates in mTeSR1 medium (StemCell Technologies, Vancouver, Canada) at 37 °C. Cardiomyocyte differentiation was performed by modulating Wnt/β-catenin signaling, applying small molecules as previously described by Lian et al. [47]. iPSCs were dissociated mechanically or enzymatically with Versene solution (Invitrogen, Life Technologies, Woburn, MA, USA) and were seeded in 6-well or 12-well plates containing mTeSR1 medium supplemented in some cases with 5 μmol/L ROCK inhibitor (Cayman Chemical, Ann Arbor, MI, USA). Cells were maintained for additional 1–4 days (with mTeSR1 medium replaced every 1–3 days) before the beginning the differentiation protocol (day 0). On day 0, medium was changed to RPMI supplemented with B27 minus insulin (In vitrogen, Life Technologies, Woburn, MA, USA) and 8 or 10 μmol/L CHIR99021. On the next day (day 1), the medium was changed to RPMI supplemented with B27 minus insulin. On day 3, the medium was changed to RPMI supplemented with B27 minus insulin and 5 or 10 μmol/L IWP-4. On day 5, the medium was changed to RPMI supplemented with B27 minus insulin. From day 7 onwards, cells were cultured in RPMI supplemented with B27 complete supplement (Invitrogen), and medium was replaced 2–3 times a week.

### 4.9. Action Potentials, I_f_ and I_Ca,L_ Recording and Analysis

iPSC-CMs were mechanically or enzymatically (0.25% trypsin-EDTA, Biological Industries, Beit-Haemek, Israel) dissociated and plated on Matrigel-coated glass coverslips (13 mm diameter) in 24-well plates. The coverslips were incubated at 37 °C and the medium was replaced 1–2 times a week. A recovery period of at least two days after plating was allowed before performing the electrophysiological experiments. For action potential recordings, the coverslips were perfused at 37 °C with Tyrode’s solution containing (in mM): 140 NaCl, 5.4 KCl, 1.8 CaCl_2_, 1 MgCl_2_, 10 glucose and 10 HEPES titrated to pH 7.4 with NaOH. The patch pipette solution contained (mM): 120 KCl, 1 MgCl_2_, 3 Mg-ATP, 10 HEPES, and 10 EGTA titrated to pH 7.2 with KOH and adjusted at 290 mOsm with saccharose. Microelectrodes were pulled from borosilicate glass capillaries (Harvard Apparatus, Holliston, MA, USA). The following parameters were analyzed with MATLAB software: AP firing rate, MDP, dV/dt_max_, APA, AP peak, APD at 20% repolarization (APD_20_), APD_50_, and APD_90_. The following formulas were used for the calculation of rate-corrected APD_90_ (RR was replaced dwith 60/beat per minute (BPM)): Bazett (APD_90_/(60/BPM)^1/2^), Fridericia (ADP_90_/(60/BPM)^1/3^), Hodges (ADP_90_ + 1.75 × (beat rate − 60)) and Framingham (ADP_90_ + 0.154 × (1 − (60/BPM))). Recordings of ~170 s of cardiomyocytes’ spontaneous activity were used for the search and detection of afterdepolarization event/s. Afterdepolarization was defined as: (1) DAD—abnormal depolarization occurring after the completion of the AP repolarization with an amplitude ≥3% of the preceding APA; (2) EAD—abnormal depolarization occurring before the completion of the AP repolarization with an amplitude ≥3% of the preceding APA [48]. For beat rate variability (BRV) analysis, ~100 s of cardiomyocyte spontaneous activity was used and IBIs were calculated by using the MATLAB software. The β-adrenergic response was tested in iPSC-CMs using increasing concentrations (10^−9^–10^−6^ M) of isoproterenol or until cessation of spontaneous firing occurred. Positive chronotropic response was defined as increased beat rate in at least one concentration of isoproterenol compared to baseline. The trains protocol consisted of 4 trains (each consisted of 20 stimuli) at increasing frequencies (0.5 Hz, 1 Hz, 1.5 Hz and 2 Hz), each followed by a 20-s period without stimulation. As most of the control cardiomyocytes failed to be paced at 0.5 Hz due to their faster spontaneous firing rate, quantification of cells showing DAD event/s, arrhythmia/s or cessation of AP firing was performed only for the post-pacing intervals following the 1, 1.5 and 2 Hz trains. The pacemaker current (I_f_) was recorded from iPSC-CMs with the same Tyrode’s solution as described above, in addition to 500 μM BaCl_2_ to block I_K1_. Membrane capacitance and series resistance values were set initially after the whole-cell seal, and were adjusted and determined after series membrane resistance compensation of ~80%. The membrane was clamped at 15-s intervals, from a holding potential of −40 mV to −120 mV in 10 mV steps for 2-s pulse durations. For I_Ca,L_ recordings, membrane capacitance was determined after the whole-cell seal and series resistance was adjusted and determined after compensation of ~80%. The membrane was clamped at 200 ms depolarizing steps, from a holding potential of −70 mV ranging from −40 to +50 mV after a 20 ms −40 mV pulse. Axopatch 200B, Digidata 1322 or 1440 and pClamp10 (Molecular Devices, Sunnyvale, CA, USA), routinely used in our lab [49], were used for data amplification, acquisition and analysis. Signals were digitized at 4–10 kHz.

### 4.10. Micro-Electrode-Array (MEA) Recordings

Extracellular electrograms were recorded from spontaneously contracting iPSC-CM clusters using the MEA apparatus (Multi Channels Systems, Reutlingen, Germany), routinely used in our lab [50,51]. Recordings were performed using a sampling frequency of 1000 Hz, down-sampled to 200 Hz.

### 4.11. Measurements of Intracellular Ca^2+^ Transients

Intracellular Ca^2+^ ([Ca^2+^]_i_) transients were recorded from iPSC-CM clusters using the IonOptix Calcium and Contractility system (IonOptix LLC, Westwood, MA, USA), routinely used in our lab [23,52,53]. In brief, iPSC-CM clusters were mechanically dissected and adhered to 18 mm diameter Matrigel-coated glass slides. On the day of the experiment, slides were stained with fura-2 (2.5 μM) and were transferred to a chamber mounted on the stage of an inverted microscope and perfused at a rate of 1–1.5 mL/min Tyrode’s solution at 37 °C. The Tyrode’s solution contained: (mmol/L): 140 NaCl, 5.4 KCl, 1 MgCl_2_, 2 sodium pyruvate, 1 CaCl_2_, 10 HEPES, 10 glucose (pH 7.4 adjusted with NaOH). The clusters were paced at 0.5–2.5 Hz which corresponded to a frequency 20–50% higher than the spontaneous beating rate. Analysis was performed using the IonOptix designated system. RyR-mediated SR Ca^2+^ release was measured by a brief application of caffeine (10 mM). The caffeine response was quantified by calculating 3 parameters: (1) recovery time—the time from the peak of caffeine-induced [Ca^2+^]_i_ rise to the first [Ca^2+^]_i_ transient; (2) the percent change in caffeine-induced [Ca^2+^]_i_ response amplitude, compared to pre-caffeine amplitude; (3) the percent change in caffeine-induced [Ca^2+^]_i_ response area, compared to the pre-caffeine value.

### 4.12. Transmission Electron Microscopy (TEM)

Transmission electron microscopy (TEM) was performed on iPSC-CM clusters fixed with 2.5% glutaraldehyde in 0.1 M cacodylate buffer (pH 7.4, room temperature). The samples were post-fixed for 1 h in buffered 1% OsO_4_ with 1.5% K_4_Fe(CN)6 (potassium ferrocyanide-reduced osmium) in 0.1 M cacodylate buffer at room temperature. Fixed iPSC-CM clusters were embedded in 1% agar, dehydrated in graded ethanol series and embedded in epoxy resin (Agar100 resin) at 60 °C for 48 h. The ultra-thin sections were cut with a diamond knife at 60 nm thicknesses using a Leica EMUC7 ultramicrotome and double-stained with 1% uranyl acetate and Reynolds lead citrate. The ultrastructural examination was performed with a Morgagni 268 TEM (FEI Company, Eindhoven, The Netherlands) at 80 kV. Digital electron micrographs were recorded with a MegaView III CCD and iTEM-SIS software (Olympus, Soft Imaging System GmbH, Münster, Germany) was used for morphometry. Three distinct iPSC-CM clusters for each sample (control (FSE-5 m), father and son) were selected and sectioned for TEM analysis. The nuclei of iPSC-CMs were photographed under TEM microscope at 7100 magnification step. At least 25 images with nuclei having visible nucleolus in the section plane were selected for morphometry. Using AnalySIS software (Soft Imaging Systems, Olympus, Münster, Germany), the contour of the nuclei was traced for perimeter measurements and raw data recorded as Excel files.

### 4.13. RNA Sequencing

Total RNA was extracted from control (FSE-5 m and 24.5) and *LMNA*-mutated (father and son) cell pellets using the Qiacube (Qiagen, Hilden, Germany) with the RNeasy kit (cat No. 74106). A 600 μL RLT buffer (lysis buffer) was added to cell pellets of samples 24.5-1 and 24.5-2 (big pellets’ size), then 350 μL were loaded on Qiacube for automated extraction like other samples. Quality control for total RNA was performed using the TapeStation 4200 (Agilent Technologies, Santa Clara, CA, USA) with the RNA kit (cat No. 5067-5576). The RINe value of the samples was in the range of 7.9–9.6, indicating high quality. RNAseq libraries were constructed simultaneously according to the manufacture protocol (NEBNext Ultra II Directional RNA Library Prep Kit for Illumina, cat No. E7760) using 125 ng of total RNA as starting material. mRNAs pull-down was performed using the Magnetic Isolation Module (NEB, cat No. E7490). After construction, the concentration of each library was measured using Qubit (Invitrogen, Waltham, MA, USA) and the size was determined using the TapeStation 4200 with the High Sensitivity D1000 kit (cat No. 5067–5584). All libraries were mixed into a single tube with equal molarity. The RNAseq data were generated on Illumina NextSeq500, 75 cycles (single read), high-output mode (Illumina, cat No. 20024906). Quality control was assessed using Fastqc (v0.11.5), reads were trimmed for adapters, low quality 3′ and minimum length of 20 using CUTADAPT (v1.12). Seventy-five bp single-end reads were aligned to human reference genome (Homo_sapiens.GRCh38.dna.primaryassembly.fa downloaded from ENSEMBL) and annotation file (Homo_sapiens.GRCh38.92.gtf downloaded from ENSEMBL) using STAR aligner (v2.6.0a). The number of reads per gene was counted using Htseq (v0.9.1). Statistical analysis was performed using DESeq2 R package (version 1.26.0) (Genome Biology 2014 15:550). The number of reads per gene was extracted into CountWithSymbol.xlsx and NormalizedCountWithSymbol.xlsx files for raw counts and normalized counts, respectively. The similarity between samples was evaluated within DESeq2 package using correlation matrix, shown in two plots—heatmap and principal component analysis (PCA). Additionally, the differential expressed genes (DEGs) lists were imported and analyzed using the Ingenuity Pathway Analysis (IPA) software.

### 4.14. Statistical Analysis

Results are presented as mean ± SEM. The results were determined and statistically tested after outliers exclusion using: mean ± 2•SD (for beat rate, AP parameters, cAPD_90_, nuclear perimeter, COV, SD1, SD2, caffeine-induced Ca^2+^ signal amplitude, area and recovery time and current–voltage (I–V) curves of I_f_ and I_Ca,L_). The comparison between control, father and son iPSC-CMs was performed using One Way ANOVA followed by Holm–Sidak test (for beat rate, AP parameters, cAPD_90_, nuclear perimeter, COV, SD1, SD2 and caffeine-induced Ca^2+^ signal amplitude, area and recovery time) or Two-way ANOVA test followed by Holm–Sidak test (for current–voltage (I–V) curves of I_f_ and I_Ca,L_) using SigmaPlot 12.0 or 14.0 software (Systat Software International, San Jose, CA, USA). A value of *p* < 0.05 was considered statistically significant. For RNA sequencing, statistical analysis was performed using DESeq2 R package (padj < 0.05 was considered statistically significant).

## 5. Conclusions

*LMNA*-mutated cardiomyocytes displayed the following electrophysiological abnormalities: decreased spontaneous firing rate and I_f_ density, APD prolongation and increased I_Ca,L_ density, DADs, arrhythmias and increased BRV. Additionally, *LMNA*-mutated cardiomyocytes showed altered response to β-adrenergic stimulation, rapid pacing and caffeine application. Notably, pharmacological treatment with KB-R7943 eliminated DADs in *LMNA*-mutated cardiomyocytes. Finally, we found both structural and gene expression alterations, including in genes encoding for major Ca^2+^ handling proteins in *LMNA*-mutated iPSC-CMs. Overall, our study demonstrates cellular mechanisms underlying electrophysiological abnormalities of *LMNA*-related DCM, and shows the applicability of iPSC-CMs in investigating the pathophysiology of *LMNA*-related DCM which may lead to the development of novel therapies for the disease.

## Data Availability

The data presented in this study are available on request from the corresponding author.

## Supplementary Materials

The following are available online at https://www.mdpi.com/article/10.3390/ijms22157874/s1.
