# Supplementary material for: Investigating LMNA-Related Dilated Cardiomyopathy Using Human Induced Pluripotent Stem Cell-Derived Cardiomyocytes
Source: Int J Mol Sci. 2021 Jul 23;22(15):7874. doi: 10.3390/ijms22157874 (PMC8346174; doi:10.3390/ijms22157874)
Supplement: Supplementary file 1 [file ijms-22-07874-s001.zip › ijms-1287737-supplementary.pdf]

<u>**Supplemental Material**</u>

Investigating *LMNA*-related dilated cardiomyopathy using human induced pluripotent stem cell-derived cardiomyocytes

Yuval Shemer [1], Lucy N. Mekies [1], Ronen Ben Jehuda [1,2], Polina Baskin [1], Rita Shulman [1], Binyamin Eisen [1], Danielle Regev [1], Eloisa Arbustini [3], Brenda Gerull [4], Mihaela Gherghiceanu [5], Eyal Gottlieb [6], Michael Arad [7,8] and Ofer Binah [1,*]

[1] Department of Physiology, Biophysics and Systems Biology, Rappaport Faculty of Medicine and Rappaport Research Institute, Technion - Israel Institute of Technology, Haifa 31096, Israel; yyshemer@gmail.com (Y.S.); lucymekies@yahoo.fr (L.N.M.); ronenbeje@gmail.com (R.B.J.); polinabaskin@campus.technion.ac.il (P.B.); rita.shulman@gmail.com (R.S.); binyae@campus.technion.ac.il (B.E.); r.danielle@campus.technion.ac.il (D.R.); binah@technion.ac.il (O.B.)
[2] Department of Biotechnology, Technion - Israel Institute of Technology, Haifa 3200003, Israel; ronenbeje@gmail.com (R.B.J.)
[3] Centre for Inherited Cardiovascular Diseases, IRCCS Foundation, Policlinico San Matteo, Pavia 27100, Italy; e.arbustini@smatteo.pv.it (E.A.)
[4] Comprehensive Heart Failure Center and Department of Internal Medicine I, University Hospital Würzburg, Würzburg 97080, Germany; gerull_b@ukw.de (B.G.)
[5] Victor Babes National Institute of Pathology, Bucharest 050096, Romania; mgherghiceanu@yahoo.com (M.G.)
[6] Department of Cell Biology and Cancer Science, Rappaport Faculty of Medicine, Technion - Israel Institute of Technology, Haifa 31096, Israel; e.gottlieb@technion.ac.il (E.G.)
[7] Leviev Heart Center, Sheba Medical Center, Ramat Gan 52621, Israel; michael.arad@sheba.health.gov.il (M.A.)
[8] Sackler Faculty of Medicine, Tel Aviv University, Tel Aviv 6997801, Israel; michael.arad@sheba.health.gov.il (M.A.)

***** <u>**Corresponding author:**</u>

Ofer Binah, PhD

Department of Physiology, Biophysics and Systems Biology

Ruth & Bruce Rappaport Faculty of Medicine

1 Efron Street, POB 9649, Haifa, 31096 Israel

Email: binah@technion.ac.il; Tel: +972-4-8295262; Fax: +972-4-8513919

<u>Supplementary Figures and Figure Legend</u>

Supplementary Figure S1

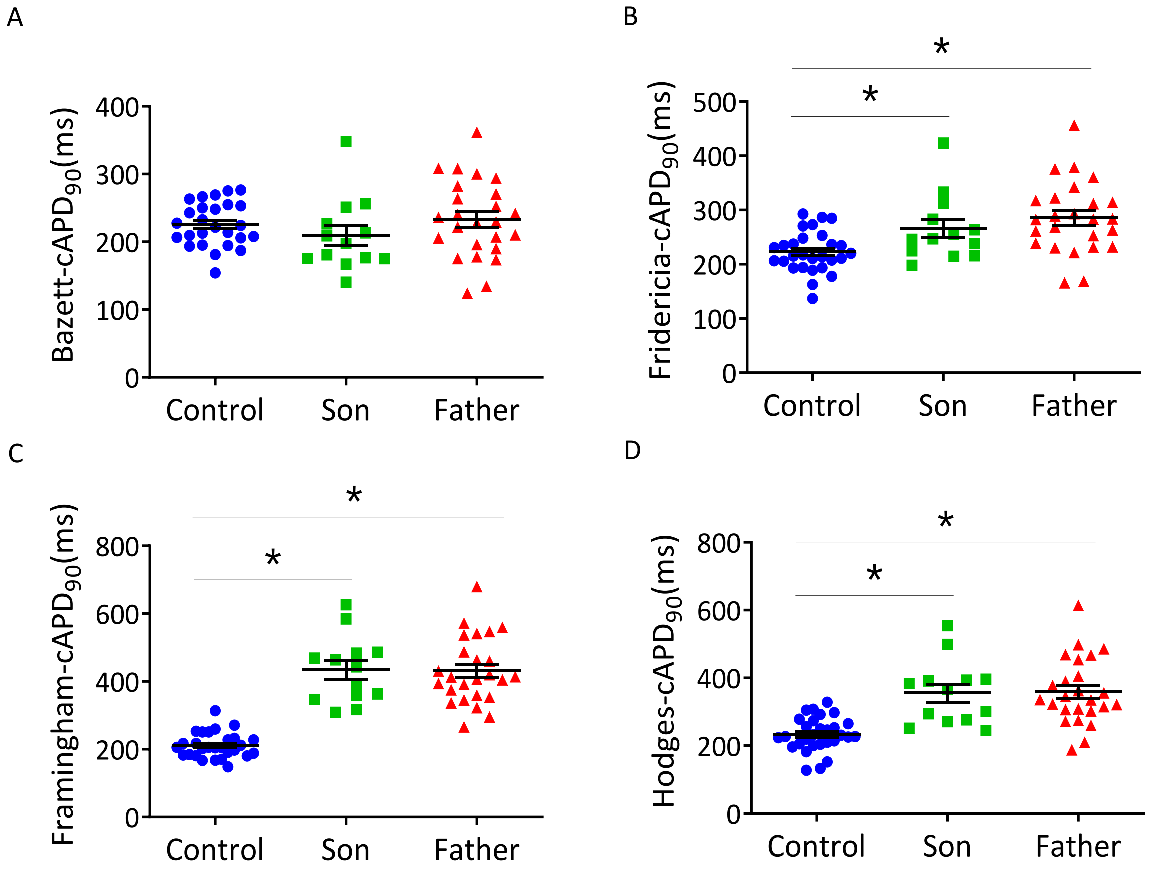

Correction of APD$_{90}$ (cAPD$_{90}$) for beat rate changes in control, and *LMNA*-mutated father and son iPSC-CMs. (A) cAPD$_{90}$ with Bazett formula. (B) cAPD$_{90}$ with Fridericia formula. (C) cAPD$_{90}$ with Framingham formula. (D) cAPD$_{90}$ with Hodges formula. Control, n=30; son, n =14; father, n=27. One-way ANOVA was performed followed by Holm-Sidak *post hoc* analysis. *p<0.05.

Supplementary Figure S2

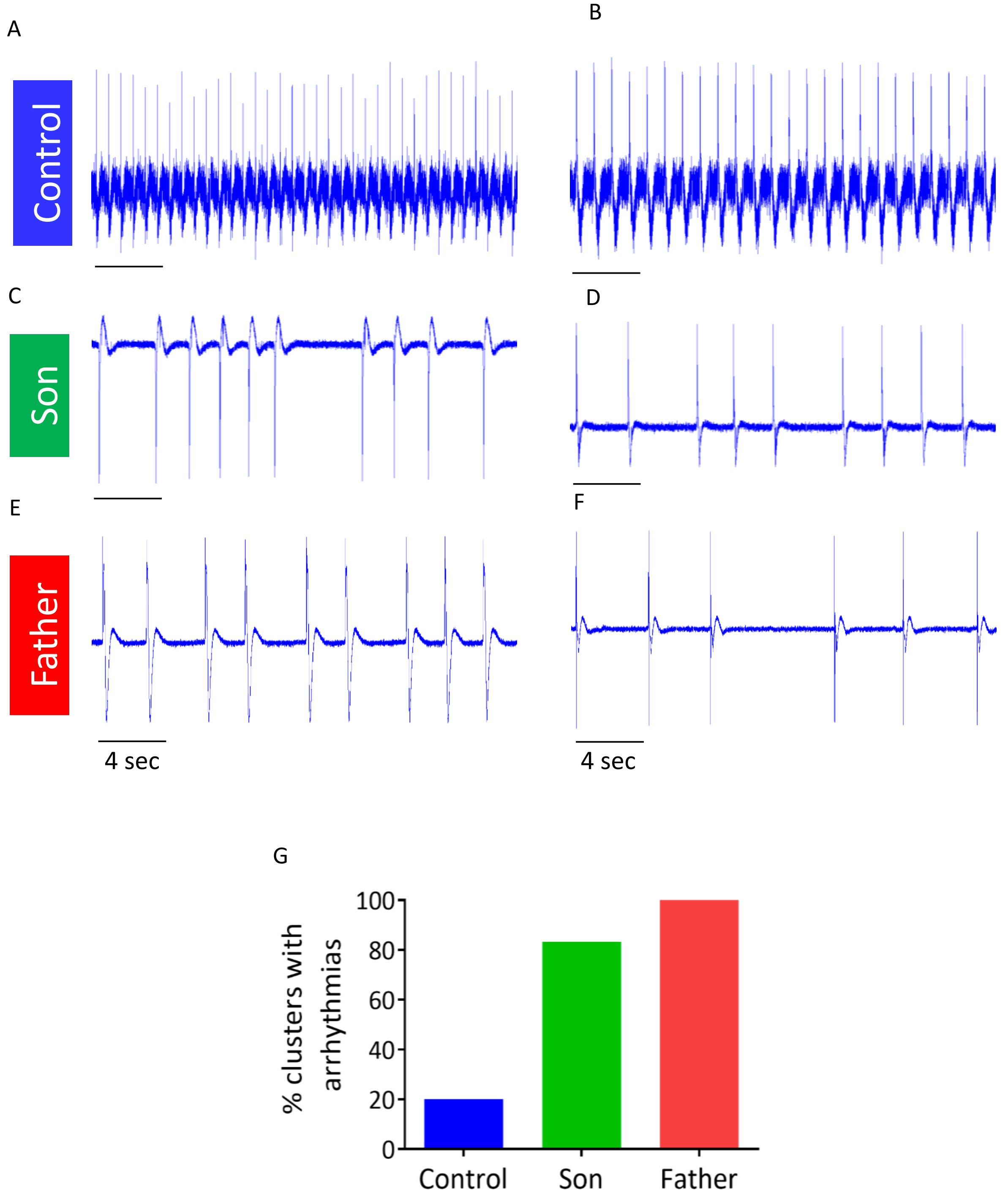

Arrhythmias in *LMNA*-mutated iPSC-CMs at the network level. (A-F) Representative recordings of extracellular electrograms from control (A, B), son (C, D) and father (E, F)

spontaneous beating clusters, using the MEA data acquisition system. Note arrhythmias in father and son iPSC-CMs. (G) Percentage of spontaneously beating clusters showing arrhythmias in control, father and son iPSC-CMs (Control, n=5; son, n=6; father, n=4).

Supplementary Figure S3

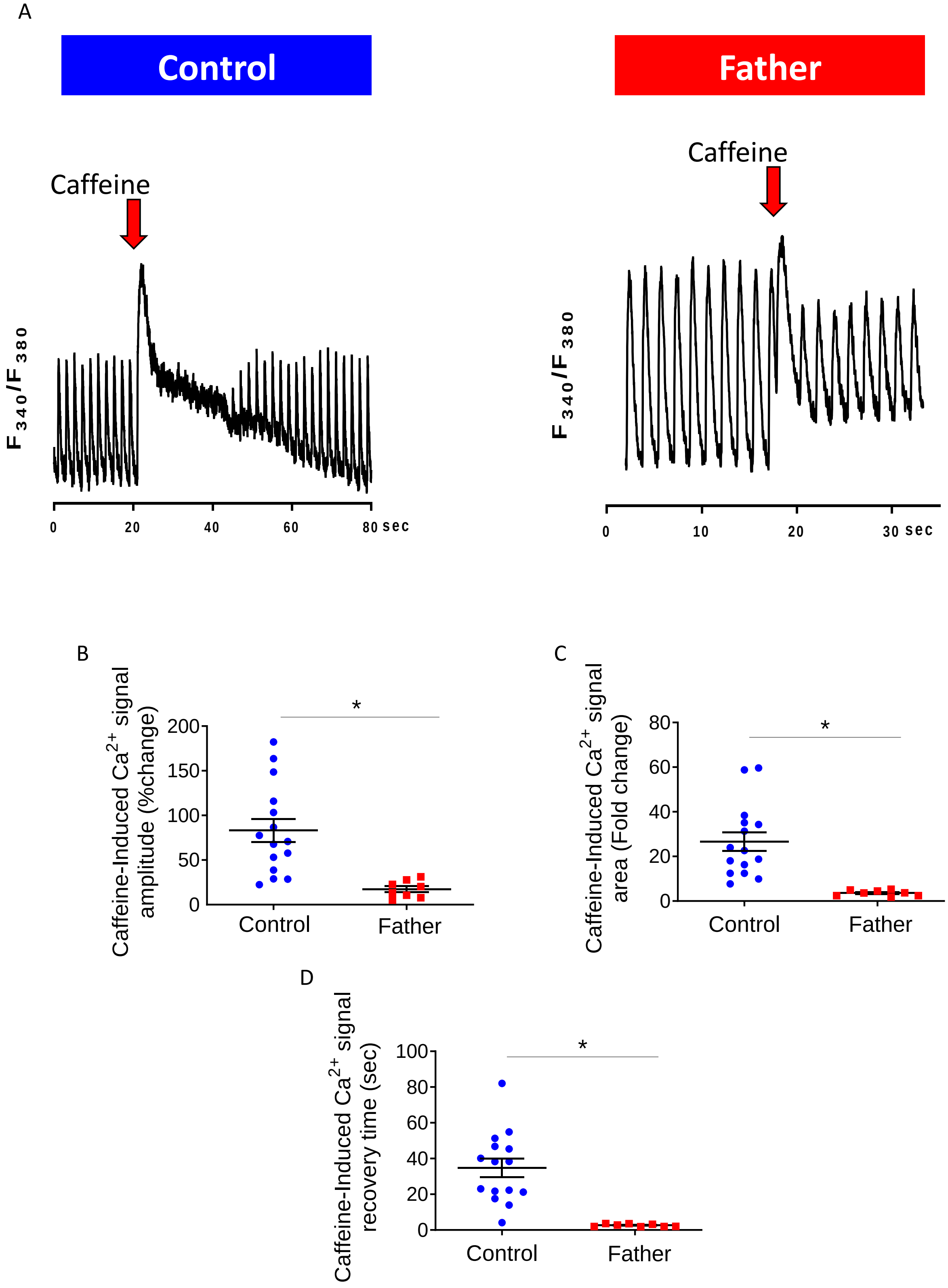

The response of $[Ca^{2+}]_i$ to caffeine (10 mM) in control and father iPSC-CMs. (A) Representative $[Ca^{2+}]_i$ transients from control and father *LMNA*-mutated iPSC-CMs

demonstrate the effect of caffeine. (B) Percent change in caffeine-induced Ca^{2+} signal amplitude compared to the pre-caffeine amplitude; (C) Percent change in area of the caffeine-induced $[Ca^{2+}]_i$ signal compared to the pre-caffeine area; (D) The mean recovery time, calculated as the time from the peak of caffeine-induced $[Ca^{2+}]_i$ rise to the first measurable $[Ca^{2+}]_i$ transient. Control, n=16; father, n=8. One-way ANOVA was performed followed by Holm-Sidak *post hoc* analysis. *$p<0.05$.

Supplementary Figure S4

A

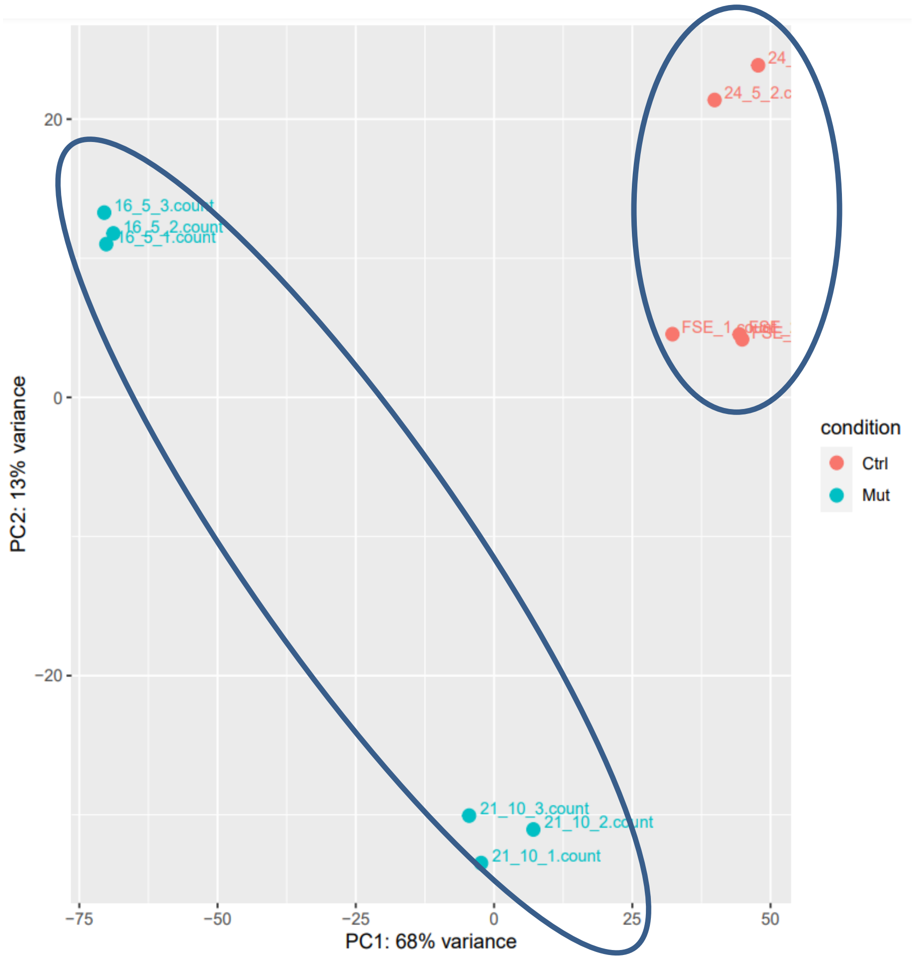

B

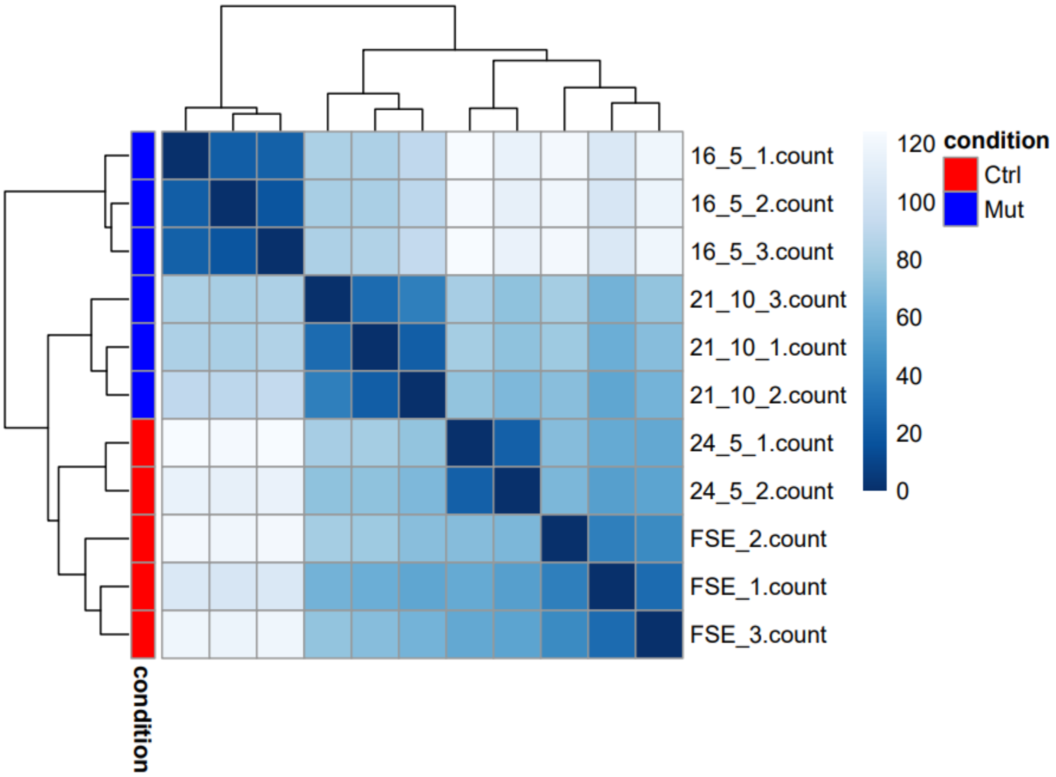
16_5_1.count
16_5_2.count
16_5_3.count
21_10_3.count
21_10_1.count
21_10_2.count
24_5_1.count
24_5_2.count
FSE_2.count
FSE_1.count
FSE_3.count
120
100
80
60
40
20
0
condition
Ctrl
Mut
condition

C

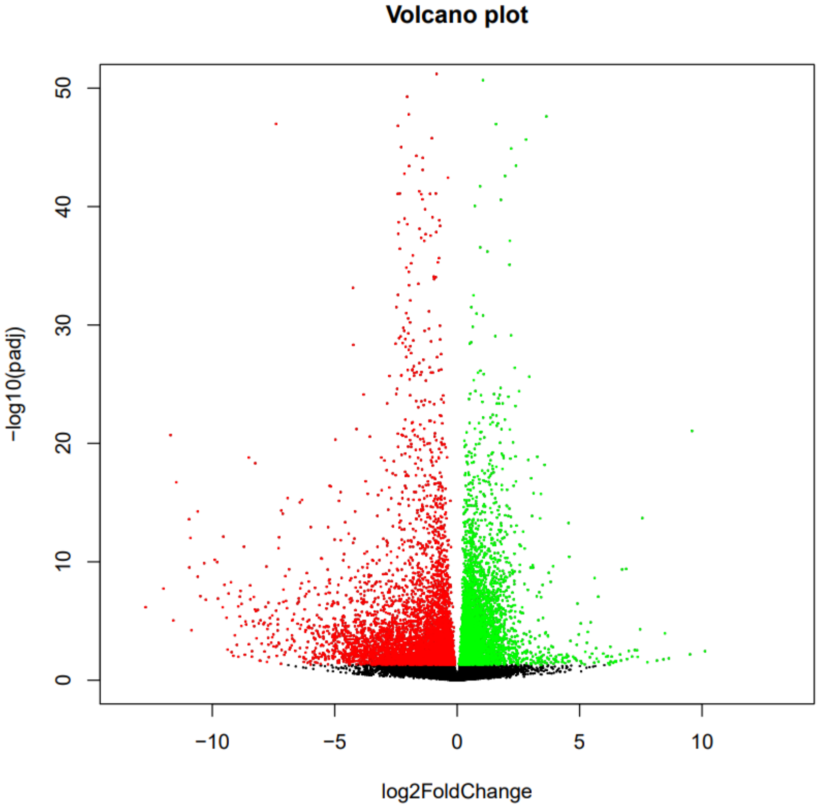
Volcano plot
−log10(padj)
0
10
20
30
40
50
−10
−5
0
5
10
log2FoldChange

D

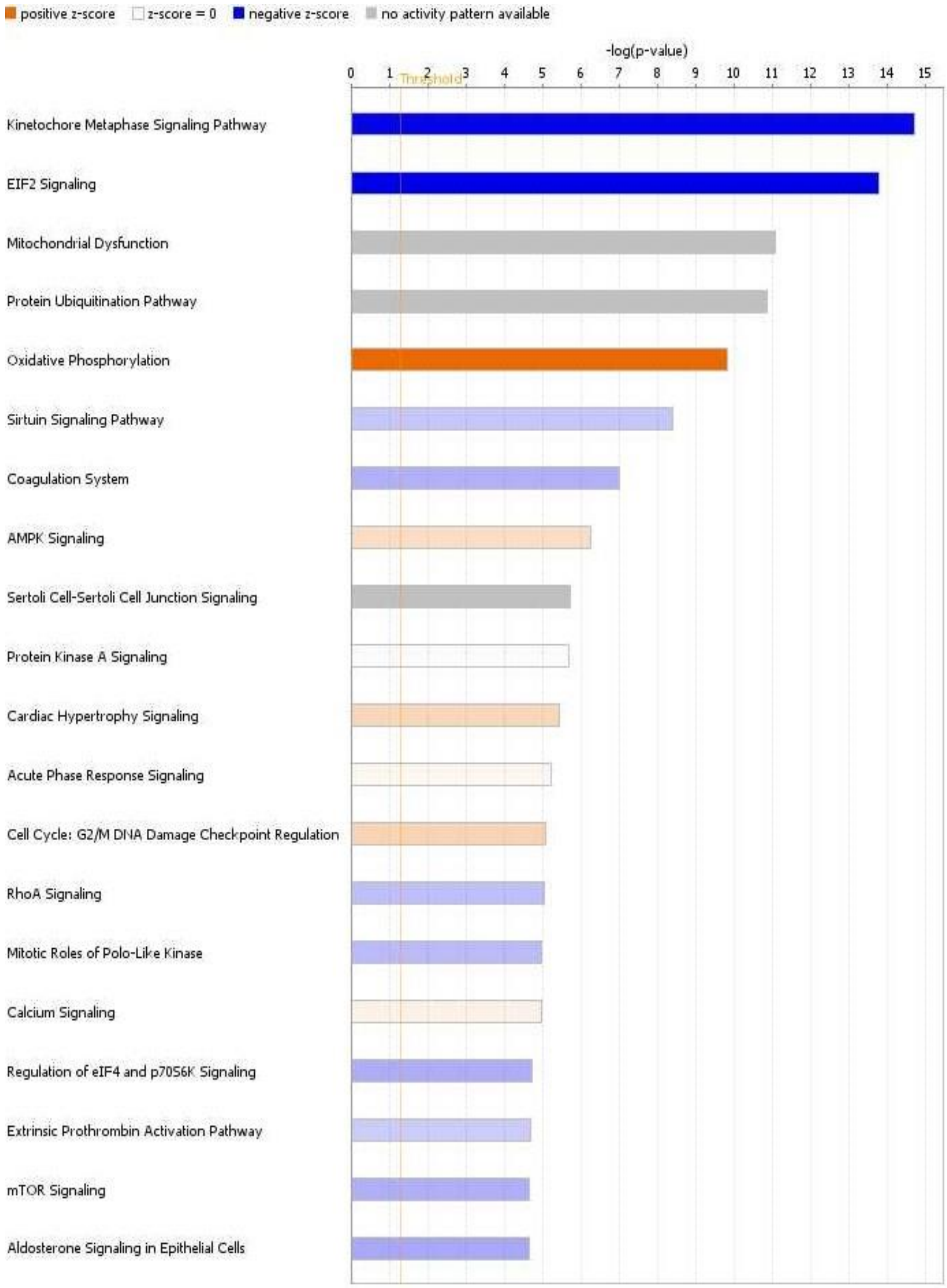

Altered gene expression in *LMNA*-mutated iPSC-CMs. (A) PCA and (B) heatmap of gene expression profile in control (clones 24.5 and FSE-5m), and father (clone 16.5) and son (clone

21.10) *LMNA*-mutated iPSC-CMs. (C) Volcano plot showing gene expression changes in *LMNA*-mutated iPSC-CMs compared to control iPSC-CMs. The x-axis shows the fold-change in gene expression between the two groups. The y-axis shows the statistical significance of the differences. The black dots represent genes without significant different expression. The green dots represent significantly upregulated genes. The red dots represent significantly downregulated genes. A total of 9,794 differentially expressed genes (DEGs) were identified (padj<0.05). (D) Ingenuity Pathway Analysis (IPA) showing the top 20 canonical pathways enriched with DEGs in *LMNA*-mutated iPSC-CMs compared to control iPSC-CMs.

Supplementary Tables

Table S1: Shared rare variants between both father and son iPSCs

Chr.	HGVS	Protein	Gene	Type	Classification[1]	ID	MAF*
1	c.1024G>A	p.(Glu342Lys)	LMNA	Missense	Likely pathogenic	-	-
3	c.233C>T	p.(Thr78Met)	CAV3	Missense	Uncertain significance	rs72546668	0.0027

Table S2: Additional rare variant only found in the father iPSCs

Chr.	HGVS	Protein	Gene	Type	Classification[1]	ID	MAF*
2	c.52384C>T	p.(Leu17462Phe)	TTN	Missense	Uncertain significance	-	-

*Genome Aggregation Database (gnomAD; https://gnomad.broadinstitute.org/) (accessed on September 2019).

Gene Transcripts: LMNA (NM_170707.3), TTN(NM_001267550.2), CAV3 (NM_033337.2).

Abbreviations: Chr., chromosome; HGVS, *Human Genome Variant Society*-nomenclature; ID, identification number of reference SNP; MAF, minor allele frequency; LMNA, lamin A/C; CAV3, caveolin-3; TTN, titin [1].

References

1. Richards, S.; Aziz, N.; Bale, S.; Bick, D.; Das, S.; Gastier-Foster, J.; Grody, W.W.; Hegde, M.; Lyon, E.; Spector, E.; Voelkerding, K.; Rehm, H.L. Standards and guidelines for the interpretation of sequence variants: a joint consensus recommendation of the American College of Medical Genetics and Genomics and the Association for Molecular Pathology. *Genet. Med.* **2015**, *17*, 405–424.
